# Efficacy of interventions for suicide and self-injury in children and adolescents: a meta-analysis

**DOI:** 10.1038/s41598-022-16567-8

**Published:** 2022-07-19

**Authors:** Lauren M. Harris, Xieyining Huang, Kensie M. Funsch, Kathryn R. Fox, Jessica D. Ribeiro

**Affiliations:** 1 Department of Psychology, Florida State University, 1107 W. Call St., Tallahassee, FL 32306 USA; 2 School of Behavioral and Brain Sciences, University of Texas at Dallas, Richardson, TX USA; 3 Department of Psychology, University of Denver, Denver, CO USA

**Keywords:** Psychology, Paediatrics

## Abstract

Despite increased numbers of children and adolescents seeking and receiving mental health treatment, rates of self-injurious thoughts and behaviors (SITBs) in youth are rising. In the hopes of aiding ongoing efforts to alleviate the burden of SITBs in this vulnerable population, the present study summarizes current knowledge on the efficacy of SITB interventions in children and adolescents. We conducted a meta-analysis of randomized controlled trials (RCTs) assessing treatment effects on SITBs in child and adolescent populations. A total of 112 articles comprising 558 effect sizes were included in analyses. Nearly all interventions produced nonsignificant reductions in SITBs. For binary SITB outcomes, a nonsignificant treatment effect was detected, with an RR of 1.06 (95% CIs [0.99, 1.14]). For continuous SITB outcomes, analyses also yielded a nonsignificant treatment effect (g = − 0.04 [− 0.12, 0.05]). These patterns were largely consistent across SITB outcomes, regardless of intervention type, treatment components, sample and study characteristics, and publication year. Our findings highlight opportunities for improving SITB intervention development and implementation in child and adolescent populations. The most efficacious interventions are likely to directly target the causes of SITBs; therefore, future research is needed to identify the causal processes underlying the onset and maintenance of SITBs in youth.

## Introduction

Despite increased numbers of children and adolescents seeking and receiving mental health treatment^1,2^, rates of self-injurious thoughts and behaviors (SITBs) in youth are rising^3,4^. Major scientific efforts have been dedicated to mitigating this significant public health issue, but sustained reductions in youth SITBs have not yet been achieved. Changing this concerning trajectory necessitates the identification and dissemination of highly efficacious interventions to address SITBs in child and adolescent populations.

Over the last several decades, there has been growing recognition within the scientific community of the unique treatment needs of children and adolescents^5,6^. Rather than conceptualizing youth as a transient period of normative “storm and stress,” researchers have turned their attention to better understanding the unique mechanisms that may contribute to the onset and maintenance of psychopathology during this critical developmental period^7^. This has led to a proliferation of studies exploring the utility of developing mental health interventions specifically tailored for child and adolescent populations^8^. While several interventions have garnered strong empirical support for addressing a variety of mental disorders in youth, including depression^9^, anxiety^10^, and substance use^11^, there is currently no gold-standard treatment for youth SITBs.

Prior systematic and meta-analytic reviews have sought to identify the most efficacious youth SITB interventions by summarizing findings from existing intervention trials. Notably, most existing efforts have been circumscribed to either particular intervention categories (e.g., psychosocial, pharmacological), intervention types (e.g., cognitive-behavioral therapy [CBT], dialectical behavior therapy [DBT], antidepressant medication), and/or SITB outcomes (e.g., nonsuicidal self-injury [NSSI], suicidal behaviors, self-harm^12–20^). Although these summaries have provided valuable insights into the current state of knowledge on youth SITB intervention techniques, most have concluded that additional research is needed to corroborate initial encouraging findings for potentially promising interventions. As such, the most efficacious interventions for youth SITBs have not yet been firmly established.

To address this knowledge gap, the purpose of the present meta-analysis is to significantly advance knowledge of the efficacy of youth SITB interventions. To this end, we update and extend a recent large-scale meta-analytic effort designed to evaluate SITB intervention efficacy across all age groups^21^. In this broad meta-analysis, Fox and colleagues addressed several major questions about both psychosocial and pharmacological SITB interventions, such as *What is the overall efficacy of SITB interventions?* and *Are some SITB interventions better than others?* Given the broad scope of this study, however, specific questions pertaining to child and adolescent populations were not addressed. By focusing exclusively on data drawn from efficacy trials conducted in child and adolescent populations, the present study will address more fine-grained questions about factors that may influence treatment efficacy in youth, such as *Do interventions developed for children and adolescents produce greater reductions in SITBs than interventions created for adults?* and *Do efficacious psychosocial interventions for youth share common “active ingredients” which contribute to SITB reduction?* We expect that narrowing our focus to children and adolescents will allow us to establish not only which interventions are most efficacious for this population, but also the conditions under which the best treatment outcomes may be achieved. By contextualizing our findings within the broader youth SITB treatment literature, we aim to shed light on opportunities for improvement in the way that SITB interventions are developed and implemented in child and adolescent populations. Below, we specify our major questions of interest.

### Published literature and overall efficacy

The literature examining SITB intervention efficacy in youth has grown substantially^17,22^. To provide insight into commonly evaluated interventions, frequently targeted SITBs, and changes in the literature examining SITB intervention efficacy in youth, we examine the number of papers and effect sizes: (a) over time, (b) for each SITB outcome of interest, and (c) for each intervention evaluated with at least one randomized controlled trial (RCT). We then assess overall treatment efficacy at reducing SITBs. We focus on published RCTs because they represent the most rigorously evaluated publicly available sources^23–25^.

The SITB literature lacks standardized definitions. We adhere to the convention that SITB outcomes can be dichotomized based on presence or absence of suicidal intent. Intentional self-directed harm without suicidal intent will be considered NSSI. When information regarding suicidal intent is not provided, we will classify outcomes as self-harm. Suicide-related cognitions and plans will be considered suicidal ideation; self-directed harm with intent to die will be considered a suicide attempt. Death from self-directed behaviors with suicidal intent will be classified as suicide death. If other SITB outcomes are not specified, we will assess the effects of treatment on SITB-related hospitalization.

### Moderators of treatment efficacy

#### Treatment type

To inform treatment and prevention efforts, it is necessary to evaluate whether certain interventions are more efficacious at reducing SITBs than others. We hypothesize that most interventions would produce small, significant reductions in SITBs, with treatments specifically designed to target SITBs producing slightly greater reductions^17,21,26^.

#### Publication year or decade

We hypothesize that new studies have built upon prior research, resulting in improved treatment efficacy. We examine whether trajectories of efficacy of certain treatments are driving improvement or stagnation in overall efficacy.

#### Treatment target

Evidence is mixed regarding whether interventions designed to target other outcomes (e.g., mental disorders) are sufficient to reduce SITBs^27^. We will evaluate whether primary treatment target influences intervention efficacy. If there is no benefit to directly targeting SITBs, this would suggest that resources should be directed toward treatments that are efficacious for the most outcomes. Targeted treatments should be prioritized if there is an advantage to directly targeting SITBs.

#### Sample and study characteristics

It is unknown whether sample characteristics such as average age, clinical severity, sex, and race moderate treatment efficacy. We examine whether there are groups who may benefit most from treatment and identify groups who may require more tailored interventions.

We anticipated consistency across included RCTs; nevertheless, nuanced differences in design and quality may moderate treatment efficacy. We hypothesized that active interventions compared to no treatment would yield stronger treatment effects than those compared to other active treatments or placebo, which would indicate a beneficial influence of nonspecific treatment elements. We further hypothesized that RCTs which leverage systematic procedures for randomization and intervention delivery would yield the strongest, most reliable efficacy estimates. Similarly, we expected that RCTs requiring preintervention training and treatment adherence checks for therapists would produce stronger treatment effects than those which did not.

#### Target population

While some SITB interventions have been adapted for youth, others have been tested in, but not specifically tailored for, child/adolescent populations. Interventions which consider age may be better equipped to target unique developmental and environmental risk factors^28,29^. We test whether an intervention’s target population moderates treatment efficacy.

#### Common treatment components

It has been hypothesized that efficacious psychosocial interventions for youth share “active ingredients” which contribute to SITB reduction. Evaluating whether treatment components outlined in prior studies^13,17,22^ moderate overall treatment efficacy may elucidate common mechanisms of change across interventions.

#### Pharmacotherapy and medication type

In 2004, the Food and Drug Administration warned that antidepressant medications may cause new or increased suicidality in adolescents^30^. These recommendations were based on meta-analytic findings linking antidepressant use and suicidality^31,32^. By contrast, epidemiological data indicates that increased antidepressant use in youth is inversely correlated with suicide rates^16,33^. Given the detrimental consequences of untreated depression^34^, determining potential iatrogenic effects of pharmacological interventions is critical for advancing knowledge of SITB treatment efficacy.

No medications are designed to address SITBs; rather, existing medications target psychiatric conditions that may include SITBs as symptoms or sequelae^35^. Because SITBs are often monitored in treatment studies as adverse events, the effects of pharmacological interventions on SITBs can be quantified. Clarifying the effects of specific medications on SITB outcomes can inform safe and effective treatment of youth SITBs.

Through addressing these questions, the present meta-analysis aims to address critical gaps in our knowledge of the efficacy of youth SITB interventions, facilitate efforts to reduce the burden of SITBs in youth, and provide recommendations for future research.

## Method

### Literature search

This meta-analysis updates and extends a broader meta-analytic effort^21^ evaluating SITB intervention efficacy across age groups to provide more refined results specific to children and adolescents. The original literature search identified RCTs published before January 1, 2018, in PubMed, PsycINFO, and Google Scholar. Additional studies were added through a search of ClinicalTrials.gov and references sections of reviews/meta-analyses found within these databases. Search terms included permutations of “treatment,” (i.e., “treatment,” “intervention,” “therap*,”) crossed with permutations of “suicide” and “self-injury” (i.e., “suicid*,” “self-injur*,” “self-directed violence,” “self-harm,” “self-mutilation,” “self-cutting,” “self-burning,” “self-poisoning”). The same search strategy was used for each database. Broad search terms were intentionally retained for the original study to ensure that no potentially relevant articles were missed.

**Figure 1:**
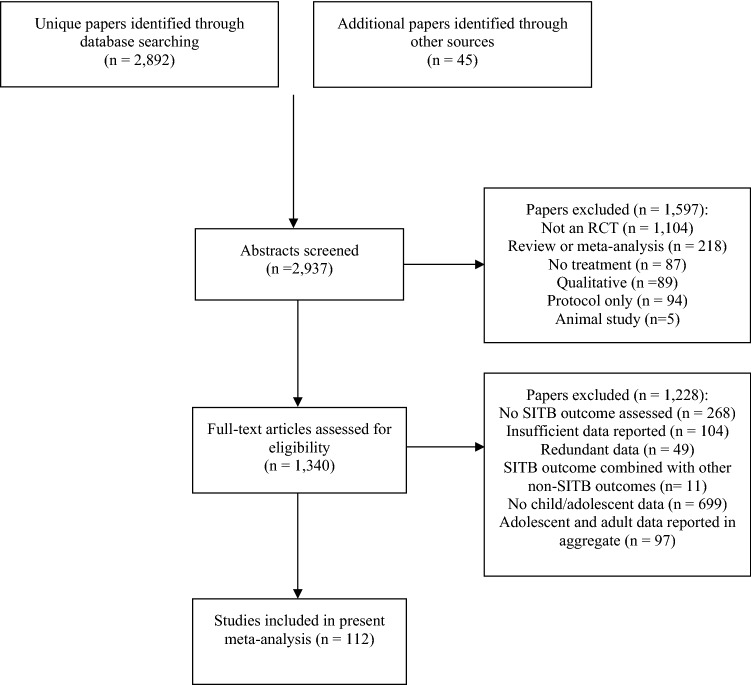
PRISMA diagram.

We updated this search to include studies published before January 1, 2022. Because we were specifically interested in RCTs conducted in child and/or adolescent populations, we added additional terms (e.g., “child,” “adolescent,” “randomized”) to streamline our search of newly published articles [Our entire search string for the updated search was as follows: (treatment OR intervention OR therapy) AND (suicide OR self-injury OR self-directed violence OR self-harm OR self-mutilation OR self-cutting OR self-burning OR self-poisoning) AND (child OR adolescent OR youth) AND (randomized OR RCT)]. In total, of 2892 potentially qualifying papers, 112 were retained (Fig. 1).

### Inclusion and exclusion criteria

Only RCTs with participants under age 18 at the start of the study were included. Studies were required to randomly assign participants to a treatment or control condition and assess SITBs at post-treatment. Because we were interested in effects on SITB occurrence, severity, and frequency, studies that assessed related or composite outcomes (e.g., attitudes towards SITBs, alcohol use with concurrent NSSI) were excluded. Studies were also excluded if they were unavailable in English.

Studies that did not include necessary statistical information (i.e., sufficient data to calculate either a risk ratio or Hedges’ *g*) after contacting corresponding authors were also excluded. When effect sizes were not reported as either risk ratios or Hedges’ *g*, they were converted using the *metafor* package in R. The following data structures were able to be converted: 2 × 2 tables with rates or number of events, chi-squared analyses, Cohen’s *d* and its variants; *t-*tests and their variants, odds ratios, and rate ratios.

### Data extraction and coding

Studies included in the original meta-analysis were coded by authors and research assistants. All coders underwent accuracy training and were required to demonstrate a high level of accuracy before beginning official coding procedures. All codes were completed via an iterative process, wherein initial codes were completed by a co-author with an advanced degree in psychology, then checked for accuracy by two independent second coders with advanced degrees in psychology. All discrepancies were discussed and resolved by consensus among all authors, such that all authors agreed upon the final version of each code. Statistics were extracted from individual RCTs whenever possible; otherwise, aggregate statistics were extracted.

#### Author, year, and decade codes

The author and publication year/decade for each manuscript were recorded.

#### SITB outcome

The following codes were used to designate various SITB-related outcomes: (1) nonsuicidal self-injury (NSSI; i.e., intentional self-directed harm in the absence of suicidal intent), (2) self-harm (i.e., intentional self-harm where information regarding suicidal intent was not assessed or provided), (3) suicide ideation (i.e., cognitions related to suicide, including suicide plans), (4) suicide attempt (i.e., self-directed harm with at least some intent to die), (5) suicide death (i.e., any death resulting from self-directed behaviors with suicidal intent), (6) SITB-related psychiatric hospitalizations or hospital visits, and (7) other/combined SITBs.

#### Sample severity

Samples were coded as “general” if participants were recruited from the community without being required to have a history of psychopathology or SITBs, “clinical” if they were recruited for a history of psychopathology, and “SITB” if participants were recruited based on a history of some form of SITB.

#### Sample characteristics

The following sample characteristics were recorded when available: (1) average age, (2) age group (i.e., children [average age of sample < 10], adolescents [average age of sample ≥ 10], (3) percentage of male and female participants, (4) percentage of white and non-white participants.

#### Treatment target

Treatments were coded based on primary target: (1) mental disorder, (2) SITB, or (3) other.

#### Intervention type

Intervention type codes included acute psychiatric services, case management services, Cognitive Therapy/Cognitive Behavioral Therapy (CT/CBT), Dialectical Behavior Therapy (DBT), eclectic psychotherapy (i.e., interventions that used a broad range of therapeutic modalities), family-based therapy, HIV prevention, Interpersonal Psychotherapy, medication only, mindfulness/meditation, psychiatric medication combination treatment (i.e., concurrent psychosocial and pharmacological treatment), parenting skills training, partial hospitalization, psychoanalysis/insight-based therapy, psychoeducation, safety planning/means restriction, and suicide prevention programs.

#### Control group type

Control groups were designated as either (1) no treatment/waitlist, (2) placebo, or (3) active treatment.

#### Medication class

Medication class was coded for all interventions which included a medication component. Codes included: (1) typical antipsychotic, (2) atypical antipsychotic, (3) anxiolytic, (4) stimulant, (5) selective serotonin reuptake inhibitor (SSRI), (6) selective norepinephrine reuptake inhibitor (SNRI), (7) monoamine oxidase inhibitor (MAOI), (8) tricyclic antidepressant, (9) noradrenergic and specific serotonergic antidepressant (NaSSA), (10) atypical antidepressant, and (11) combination of classes.

#### Treatment characteristics and components

Treatments were coded based on several characteristics and components. Duration was coded based on total weeks of treatment. Interventions were also coded based on the inclusion of individual, family, and/or group components (i.e., individual only, family only, group only, individual and family, individual and group, family and group, or a combination of all three), whether the intervention was school based, whether individual skills training was provided, and whether the treatment was specifically designed or adapted for child or adolescent populations.

#### Study quality

Study quality was evaluated using the Quality Assessment Tool for Quantitative Studies^37^, as recommended by the *Cochrane Handbook for Systematic Reviews of Interventions*^34^. All studies were categorized as either *weak, moderate*, or *strong* based on performance in the following domains: blinding, confounders, data collection procedures, intervention integrity, selection bias, study design, and withdrawals/dropouts.

#### Therapist training and adherence

For psychosocial interventions, studies were coded based on whether therapists were required to receive pretreatment training, as well as the presence or absence of adherence checks.

### Statistical analyses

Analyses were conducted using R^36^ with the *metafor* package^37^. We used random-effects models for meta-analyses, as we anticipated high between-study heterogeneity (quantified using *I*^2^ tests). First, a pooled meta-analysis was conducted to examine treatment effects on all SITB outcomes. Subsequent analyses were conducted to examine effects on each SITB outcome. To ensure reliability and accuracy, we did not conduct analyses comprising fewer than five effect sizes^38^. A post-hoc power analysis was conducted using *metapoweR*^39^. Publication bias was examined using Classic and Orwin’s Fail-Safe *N*, Begg and Mazumdar Rank Correlation Test, Egger’s Regression Test, inspection of funnel plot symmetry, and Duval and Tweedie’s Trim and Fill Test. Continuous and binary outcomes were analyzed separately to ensure effect sizes shared the same meaning and scale^40^. Hedges’ *g* effect sizes were used for continuous outcomes^41^, and risk ratios (RRs) were used for binary outcomes.

Moderator analyses were conducted for all outcomes combined and subsequently for each SITB outcome. Intervention effects were examined at post-treatment based on prior meta-analytic evidence indicating that mental health treatments in youth exert the strongest effects immediately following treatment^42^.

## Results

Analyses included 558 effect sizes from 112 papers and 110 unique RCTs. See Table 1 for full descriptive results. Effects including zero events in both the treatment and control group (n = 139; 24.91%) were not included due to insufficient variance to estimate a treatment effect. Most excluded effects (n = 111; 79.86%) were drawn from studies examining medication-only interventions, followed by interventions which combined medication and psychotherapy (n = 17; 12.23%). Zero events in both groups occurred most frequently in studies examining effects on aggregate measures of SITBs (n = 40; 28.78%) or on suicide death (n = 34; 24.46%).

**Table 1:** Descriptive statistics for included studies.

Descriptive statistics by study	Mean	
Age (years)	13.56 ± 1.79	
Proportion of male/female participants	52.60%	
Treatment duration (weeks)	12.76 ± 11.35	
**Race**		
White	69.04%	
Black	18.41%	
Asian	6.65%	
Indigenous	10.35%	
Other/multiple	10.94%	

Descriptive statistics by effect size	n	%
**Decade**		
1990s	4	0.72
2000s	212	37.99
2010s	272	48.75
2020s	70	12.54
**SITB outcome**		
Suicide ideation	191	34.23
Suicide attempt	81	14.52
Suicide death	41	7.35
Self-harm	44	7.88
NSSI	37	6.63
Hospitalization	9	1.60
Other/combined SITBs	155	27.78
**Specific intervention type**		
Medication only	370	66.31
Psychotherapy and medication combined	68	12.19
CT/CBT	47	8.42
Mixed psychotherapy modalities	12	2.15
Psychoeducation	8	1.43
DBT	11	1.97
Family based therapy	7	1.26
Parenting skills training	5	0.90
HIV prevention	4	0.72
Safety planning/means restriction	4	0.72
Motivational interviewing	4	0.72
Mentalization-based therapy	3	0.53
Community mentorship program	2	0.36
Partial hospitalization	2	0.36
Psychoanalysis/insight-based therapy	2	0.36
Acute psychiatric services	1	0.18
Bullying intervention	1	0.18
Case management services	1	0.18
Interpersonal psychotherapy	1	0.18
Mindfulness/meditation	1	0.18
Prevention program	1	0.18
Protective factor approach	1	0.18
Social skills training	1	0.18
Trigeminal nerve stimulation	1	0.18
**Control group type**		
No treatment	16	2.87
Placebo	284	50.90
Active treatment	258	46.24
**Medication class**		
Selective serotonin reuptake inhibitor	109	29.46
Atypical antipsychotic	93	25.14
Selective norepinephrine reuptake inhibitor	79	21.35
Atypical antidepressant	30	8.11
Alpha-2 adrenergic agonist	14	3.78
Stimulants	11	2.97
Hypnotic	9	2.43
Combination medication	6	1.62
GirK inhibitor	6	1.62
Mood stabilizer	5	1.35
Tricyclic antidepressant	4	1.08
Noradrenergic and specific serotonergic antidepressant	2	0.54
Tricyclic antidepressants	4	1.08
**Sample severity**		
General	42	7.53
Clinical	471	84.41
SITB	45	8.06
**Age group**		
Children only	37	6.63
Children and adolescents	518	92.83
**Intervention target type**		
SITBs	94	16.85
Psychopathology	443	79.39
Other	21	3.76
**Treatment components**		
Individual only	422	75.63
Family only	11	1.97
Group only	13	2.33
Individual and family	87	15.59
Individual, family, and group	15	2.69
School-based	10	1.79
Individual skills training provided	145	77.13
Designed or adapted for adolescents	128	85.33
**Study quality**		
Weak	252	45.16
Moderate	284	50.90
Strong	22	3.94
**Therapist training and adherence**		
Therapist adherence check	151	27.06
Therapist pre-treatment training	154	27.60

### Effect estimates and publication bias

#### Overall analyses

Overall analyses for binary SITB outcomes included 362 effect sizes and yielded a nonsignificant treatment effect (RR = 1.06; 95% CIs [0.99, 1.14], *p* = 0.09). Heterogeneity across studies was low (*I*^2^ = 7.19%), and significant evidence for publication bias was not detected (funnel plots are available in Supplement 2). For continuous SITB outcomes (e.g., means/standard deviations of frequency), analyses included 50 effect sizes and yielded a nonsignificant treatment effect (*g* = − 0.04 [− 0.12, 0.05], *p* = 0.37). Heterogeneity across studies was substantial (*I*^2^ = 64.60%), and significant publication bias was not detected. See Table 2 for full results of meta-analyses.

**Table 2:** Effect sizes and publication bias across SITB outcomes.

Binary/categorical	n	RR [95% CI]	*p*	I^2^	Fail-safe N		Begg and Mazumdar rank correlation	Egger's test of intercept	Duval and Tweedie's Trim and Fill	
					Classic	Orwin's			Missing effect sizes	Adjusted RR
Overall	362	1.06 [0.99, 1.14]	0.09	7.19%	217	0	τ = − 0.01, p = 0.76	z = 1.87, p = 0.06	0	1.06 [0.99, 1.14]
Suicide ideation	126	1.03 [0.92, 1.14]	0.65	2.70%	0	0	τ = 0.04, p = 0.46	z = 0.79, p = 0.43	0	1.03 [0.92, 1.14]
Suicide attempt	53	1.21 [0.95, 1.55]	0.13	11.77%	0	0	τ = − 0.09, p = 0.35	z = 0.53 p = 0.60	0	1.21 [0.95, 1.55]
Suicide death	6	0.77 [0.47, 1.26]	0.30	0.00%	0	0	τ = 0.60, p = 0.14	z = 1.69, p = 0.09	0	0.77 [0.47, 1.26]
NSSI	28	1.18 [0.89, 1.57]	0.25	0.00%	0	31	τ = − 0.23, p = 0.09	z = − 1.25, p = 0.21	4	1.27 [0.96, 1.68]
Self-harm	30	0.99 [0.80, 1.21]	0.90	23.03%	0	0	τ = − 0.13, p = 0.34	z = 0.32, p = 0.75	0	0.99 [0.80, 1.21]
Hospitalizations	8	1.11 [0.89, 1.39]	0.33	0.00%	0	18	τ = 0.00, p = 1.00	z = − 0.85, p = 0.40	2	1.14 [0.92, 1.42]
Other/combined SITBs	111	1.16 [0.99, 1.36]	0.49	11.46%	66	0	τ = − 0.03, p = 0.60	z = 2.18, p = 0.03	0	1.16 [0.99, 1.36]

Continuous	n	Hedges’ *g* [95% CI]	*p*	I^2^	Fail-safe N		Begg and Mazumdar rank correlation	Egger's test of intercept	Duval and Tweedie's Trim and Fill	
					Classic	Orwin's			Missing effect sizes	Adjusted RR
Overall	50	− 0.04 [− 0.12, 0.05]	0.37	64.60%	5	34	τ = − 0.05, p = 0.58	z = − 1.37, p = 0.17	0	− 0.04 [− 0.12, 0.05]
Suicide ideation	33	− 0.03 [− 0.12, 0.06]	0.53	55.92%	0	0	τ = 0.003, p = 0.99	z = 0.32, p = 0.75	0	− 0.03 [− 0.12, 0.06]
Suicide attempt	3	–	–	–	–	–	–	–	–	–
Suicide death	1	–	–	–	–	–	–	*–*	–	–
NSSI	1	–	–	–	–	–	–	–	–	–
Self-harm	7	0.12 [− 0.07, 0.32]	0.22	0.00%	0	0	τ = 0.05, p = 1.00	z = 0.24, p = 0.81	0	0.12 [− 0.07, 0.32]
Hospitalizations	1	–	–	–	–	–	–	–	–	–
Other/combined SITBs	4	–	–	–	–	–	–	–	–	–

*n* number of effect sizes, *RR* weighted mean risk ratio, *CI* confidence interval, dashes indicate unavailable information; I^2^ indicates the percentage of variances due to heterogeneity between studies. Classic Fail-safe N and Orwin’s Fail-safe N represent the number of studies needed to nullify the observed effects statistically and clinically, respectively; Begg and Mazumdar Rank Correlation Test computes the rank order correlation between effect estimates and sampling variance; Egger's Test of the Intercept uses precision (i.e., the inverse of the standard error) to predict the standardized effect (i.e., effect size divided by the standard error); Duval & Tweedie's Trim & Fill estimates effect sizes after accounting for publication bias. Missing cases are the number of cases estimated to be missing below the mean. Boldface indicates significance at p < 0.05.

#### Suicide ideation

For suicide ideation, binary outcomes included 126 effect sizes and yielded a nonsignificant treatment effect of 1.03 (95% CIs [0.92, 1.14], *p* = 0.65). Heterogeneity across studies was low (*I*^2^ = 2.70%), and publication bias was not detected. For continuous outcomes, 33 effect sizes were included, again yielding a nonsignificant treatment effect (*g* = − 0.03 [− 0.12, 0.06], *p* = 0.53). Heterogeneity across studies was high (*I*^2^ = 55.92%) and significant publication bias was not detected.

#### Suicide attempt

Analyses for suicide attempt included 53 effect sizes and yielded a nonsignificant treatment effect of 1.21 (95% CIs [0.95, 1.55], *p* = 0.13). Between-study heterogeneity was low (*I*^2^ = 11.77%) and significant publication bias was not detected. An insufficient number of effect sizes were available for continuous outcomes.

#### Suicide death

Analyses for suicide death included six effect sizes and yielded a nonsignificant treatment effect of 0.77 (95% CIs [0.47, 1.26], *p* = 0.30). Between-study heterogeneity was not detected (*I*^2^ = 0.00%) and significant publication bias was not detected.

#### NSSI

Binary NSSI analyses included 28 effect sizes and yielded a nonsignificant treatment effect of 1.18 (95% CIs [0.89, 1.57], *p* = 0.25). Between-study heterogeneity was not detected (*I*^2^ = 0.00%) and significant publication bias was not detected. An insufficient number of effect sizes were available for continuous outcomes.

#### Self-harm regardless of suicidal intent

Analyses evaluating binary outcomes for self-harm included 30 effect sizes and yielded a nonsignificant treatment effect of 0.99 (95% CIs [0.80, 1.21], *p* = 0.90). Between-study heterogeneity was low (*I*^2^ = 23.03%) and significant publication bias was not detected. Analyses of continuous outcomes included seven effect sizes and yielded a nonsignificant treatment effect (*g* = 0.12 [− 0.07, 0.32, *p* = 0.22). Neither between-study heterogeneity nor significant publication bias were detected.

#### Hospitalizations

Analyses of binary hospitalization outcomes included eight effect sizes and yielded a nonsignificant treatment effect of 1.11 (95% CIs [0.89, 1.39], *p* = 0.33). Between-study heterogeneity was not detected, and publication bias was minimal. An insufficient number of effect sizes were available for continuous outcomes.

#### Other/combined SITBs

Binary analyses of other/combined SITBs included 111 effect sizes and yielded a nonsignificant treatment effect of 1.16 (95% CIs [0.99, 1.36], *p* = 0.49). Between-study heterogeneity was low (*I*^2^ = 11.46%), and publication bias was minimal. An insufficient number of effect sizes were available for continuous outcomes.

### Moderator analyses

In our moderator analyses, meta-regressions were conducted for continuous moderators; for categorical or binary moderators, we obtained separate effect estimates. See Table 3 for results of meta-regression analyses for continuous moderators, Table 4 for pooled effects results for binary and categorical moderators, and Supplement 3 for results of moderator analyses for specific SITB outcomes.

**Table 3:** Meta-regression analyses for continuous moderators across SITB outcomes.

	Publication year		Average age		Gender proportion		Treatment duration		White/non-white proportion	
	b	p	b	p	b	p	b	p	b	p
**Binary/categorical**										
Overall	− 0.01	0.16	− 0.02	0.40	− 0.001	0.64	− 0.0002	0.94	0.002	0.43
Suicide ideation	0.003	0.83	− 0.03	0.38	0.01	0.13	**0.01**	**0.03**	0.01	0.10
Suicide attempt	− 0.03	0.09	0.04	0.62	0.005	0.58	0.01	0.70	0.002	0.82
Suicide death	− 0.19	0.46	− 0.26	0.30	− 9.29	0.46	− 0.26	0.30	− 0.26	0.30
NSSI	0.02	0.66	− 0.14	0.12	− 0.01	0.45	0.02	0.23	0.01	0.44
Self-harm	− 0.02	0.14	0.03	0.82	− 0.01	0.30	− 0.01	0.41	0.01	0.60
Visits and hospitalizations	0.002	0.99	0.23	0.68	0.03	0.44	0.04	0.69	− 0.09	0.33
Other/combined SITBs	− 0.02	0.36	0.01	0.82	− 0.01	0.32	− 0.01	0.25	− 0.005	0.60
**Continuous**										
Overall	0.01	0.38	0.01	0.38	− 0.003	0.18	− 0.001	0.72	− 0.004	0.16
Suicide ideation	0.001	0.93	0.03	0.33	− 0.001	0.53	− 0.003	0.52	− 0.002	0.46
Suicide attempt	–	–	–	–	–	–	–	–	–	–
Suicide death	–	–	–	–	–	–	–	–	–	–
NSSI	–	–	–	–	–	–	–	–	–	–
Self-harm	0.04	0.12	0.25	0.23	**− 0.01**	**0.05**	**− 0.01**	**0.05**	**− **0.01	0.53
Visits and hospitalizations	–	–	–	–	–	–	–	–	–	–
Other/combined SITBs	–	–	–	–	–	–	–	–	–	–

Estimates were not reported for analyses involving fewer than five effect sizes to improve the reliability and accuracy of estimates; b indicates the regression coefficient; dashes indicate unavailable information; boldface indicates a significant effect at p < 0.05.

**Table 4:** Moderator analyses for categorical moderators.

Pooled effects	Overall			
	Binary/categorical		Continuous	
	n	RR [95% CI]	n	*g* [95% CI]
**Specific intervention type**				
Medication only	257	1.13 [1.02, 1.26]	2	–
Psychotherapy and medication combined	42	1.00 [0.84, 1.20]	9	− 0.10 [− 0.22, 0.01]
CT/CBT	26	1.15 [0.81, 1.64]	12	− 0.17 [− 0.39, 0.04]
Mixed psychotherapy modalities	4	–	8	− 0.04 [− 0.30, 0.22]
Psychoeducation	4	–	4	–
DBT	5	1.11 [0.66, 1.86]	2	–
Parenting skills training	4	–	1	–
**Control group type**				
No treatment	6	0.83 [0.69, 1.01]	8	− 0.19 [− 0.78, 0.40]
Placebo	194	1.22 [1.08, 1.38]	4	–
Active treatment	162	1.03 [0.94, 1.13]	38	− 0.01 [− 0.10, 0.07]
**Medication class**				
Selective serotonin reuptake inhibitor	95	1.20 [1.02, 1.42]	3	–
Atypical antipsychotic	51	1.05 [0.79, 1.37]	0	–
Selective norepinephrine reuptake inhibitor	58	1.18 [0.98, 1.43]	0	–
Atypical antidepressant	20	0.83 [0.46, 1.51]	0	–
Alpha-2 adrenergic agonist	10	0.97 [0.53, 1.78]	0	–
Hypnotic	5	0.64 [0.27, 1.50]	0	–
Mood stabilizer	5	0.84 [0.38, 1.87]	0	–
Combination	5	0.68 [0.12, 4.07]	0	–
**Sample severity**				
General	24	0.91 [0.79, 1.04]	7	− 0.13 [− 0.35, 0.08]
Clinical	318	1.13 [1.04, 1.23]	24	0.02 [− 0.07, 0.11]
SITB	20	0.96 [0.79, 1.17]	19	− 0.14 [− 0.37, 0.08]
**Age group**				
Children	4	–	0	–
Children and adolescents	340	1.06 [0.98, 1.14]	48	− 0.04 [− 0.13, 0.05]
**Intervention target type**				
SITBs	51	0.97 [0.86, 1.10]	28	− 0.09 [− 0.25, 0.07]
Psychopathology	300	1.12 [1.03, 1.23]	20	− 0.01 [− 0.10, 0.08]
Other	11	1.04 [0.59, 1.83]	2	–
**Treatment components**				
Individual only	285	1.12 [1.02, 1.23]	14	− 0.08 [− 0.32, 0.17]
Family only	7	1.11 [0.93, 1.31]	3	–
Group only	7	1.17 [0.98, 1.41]	5	0.003 [− 0.23, 0.23]
Individual and family	54	1.12 [0.91, 1.39]	16	− 0.07 [− 0.17, 0.02]
Individual, family, and group	3	–	8	0.11 [− 0.16, 0.38]
School–based	6	0.81 [0.64, 1.01]	4	–
Individual skills training provided	81	1.07 [0.93, 1.24]	35	− 0.06 [− 0.15, 0.03]
Designed or adapted for adolescents	88	1.01 [0.89, 1.15]	45	− 0.06 [− 0.16, 0.04]
**Study quality**				
Weak	171	1.06 [0.95, 1.17]	26	− 0.02 [− 0.13, 0.09]
Moderate	179	1.05 [0.95, 1.17]	18	− 0.09 [− 0.22, 0.04]
Strong	12	**2.57 [1.34, 4.93]**	6	0.07 [− 0.28, 0.42]
**Therapist training and adherence**				
Therapist adherence check	91	1.00 [0.89, 1.13]	35	− 0.04 [− 0.15, 0.07]
Therapist pre-treatment training	86	1.00 [0.89, 1.13]	36	− 0.02 [− 0.13, 0.08]

Estimates were not reported for analyses involving fewer than five effect sizes to improve the reliability and accuracy of estimates. *n* number of effect sizes, *RR* weighted mean risk ratio, *95% CI* 95% confidence interval. Dashes indicate unavailable information. Bold indicates an effect estimate which is significantly different from pooled effects (i.e., nonoverlapping confidence intervals).

#### Publication year

Publication year did not significantly moderate findings in pooled analyses of binary SITB outcomes or continuous SITB outcomes, or for specific SITB outcomes (Table 3).

#### Average age and age group

Neither average age of the sample nor sample age group significantly moderated findings in pooled analyses of binary SITB outcomes or continuous SITB outcomes, or for any specific SITB outcomes (Table 3).

#### Proportion female/male participants

In pooled analyses of all SITB outcomes measured continuously, but not dichotomously, we detected a marginally significant and weak moderating effect of proportion of female/male participants (*b* = − 0.01, *p* = 0.05) indicating slightly stronger effects for samples with a greater number of female participants (Table 3).

#### Treatment duration

There was no significant moderating effect of treatment duration in pooled analyses of binary SITB outcomes or continuous SITB outcomes (Table 3). A significant but weak moderating effect was detected for suicide ideation measured dichotomously (*b* = 0.01, *p* = 0.03); greater treatment duration was associated with slightly weaker treatment effects. Marginally significant and weak moderating effects were also detected for self-harm measured continuously (*b* = − 0.01, *p* = 0.05); longer treatment duration was associated with slightly greater treatment effects. Notably, all effects were weak in magnitude.

#### Proportion of white/non-white participants

There was no significant moderating effect of sample proportion of white/non-white participants in pooled analyses of binary SITB outcomes or continuous SITB outcomes, or for any specific SITB outcomes (Table 3).

#### Specific intervention type

No statistically significant moderating effects of specific intervention type were detected in pooled analyses of binary SITB outcomes or continuous SITB outcomes (Table 4). When SITB outcomes were examined separately, a significant moderating effect of medication-only was detected for other/combined SITBs; participants in the active condition were more likely to experience SITBs (RR = 1.40 [1.15, 1.70], *p* < 0.001; Table S7).

#### Control group type

No statistically significant moderating effect of control group type was detected in pooled analyses of binary SITB outcomes or categorical outcomes (Table 4). When SITB outcomes were examined separately, a significant moderating effect of control group type was detected for other/combined SITBs; studies in which control groups received placebo yielded stronger effects, such that participants in the active condition were slightly more likely to experience SITBs (RR = 1.59 [1.30, 1.94], *p* < 0.001; Table S7).

#### Medication class

No statistically significant moderating effects of medication class were detected when SITBs were measured dichotomously or continuously, either in pooled analyses (Table 4) or for specific SITB outcomes (Supplement 3).

#### Sample severity

No statistically significant moderating effects of sample severity (i.e., general, clinical, SITB) were detected when SITBs were measured dichotomously or continuously, either in pooled analyses (Table 4) or for specific SITB outcomes (Supplement 3).

#### Intervention target type

No statistically significant moderating effects of intervention target (i.e., psychopathology, SITBs, other) were detected when SITBs were measured dichotomously or continuously, either in pooled analyses (Table 4) or for specific SITB outcomes (Supplement 3).

#### Treatment components

No statistically significant moderating effects of treatment components (i.e., individual only; family only; group only; individual and family; individual, family, and group; individual skills training; adapted for children/adolescents) were detected when SITBs were measured dichotomously or continuously, either in pooled analyses (Table 4) or for specific SITB outcomes (Supplement 3).

#### Study quality

In pooled analyses of binary SITB outcomes, we detected a significant moderating effect of “strong” study quality, such that participants in the active condition were more likely to experience SITBs (RR = 2.57 [1.34, 4.93], *p* < 0.01; Table 4). No significant moderating effects of study quality were detected for pooled analyses of continuous SITB outcomes. When SITB outcomes were examined separately, a significant moderating effect of “weak” study quality was detected for self-harm, such that participants in the active arm were less likely to experience SITBs (RR = 0.78 [0.63, 0.98], *p* < 0.05).

#### Therapist training and adherence

No statistically significant moderating effects of therapist pre-treatment training or adherence checks were detected when SITBs were measured dichotomously or continuously, either in pooled analyses (Table 4) or for specific SITB outcomes (Supplement 3).

## Discussion

The aim of this meta-analysis was to evaluate SITB intervention efficacy in children and adolescents. Strikingly, we found that nearly all intervention effects were nonsignificant, despite ample power to detect even very small effects (please see Supplement 4 for post-hoc power analyses and Supplement 5 for Trial Sequential Analysis). Overall, participants in the active group of the included RCTs were about as likely to experience SITBs at post-treatment than participants in the control group. Findings were largely consistent across various SITB outcomes, types of interventions, treatment targets, sample severity, and nearly all other potential moderators. Despite increased research in recent years, intervention efficacy has not significantly improved.

These findings are consistent with emerging meta-analytic evidence on SITB intervention efficacy. The present study is an extension of a larger meta-analytic effort^21^ which found that SITB treatment effects across all age groups are small. We hoped that by narrowing our focus to child and adolescent populations, and by including more recently published studies, we might detect findings that were obscured in the larger meta-analysis. Instead, we found that SITB treatment efficacy for youth continues to fall short of even the weak treatment effects detected in the broader literature.

Most interventions examined were not originally intended to target SITBs, but rather psychopathology broadly defined. Although it is possible that there were too few studies with SITBs as an intended treatment target to detect meaningful treatment effects, we did not detect statistically significant moderating effects of treatment target. Because the causes of SITBs remain unknown^43–45^, existing SITB-specific interventions may not effectively target causal processes underlying SITBs. Identifying and disrupting these processes is critical for developing highly effective interventions.

The most common outcome was suicide ideation, which is among the least severe SITBs examined, followed by aggregate or nonspecific measures of SITBs. Due to an insufficient number of effect sizes, we were unable to evaluate most moderators for NSSI, as well as severe outcomes such as hospitalizations, suicide attempts, and suicide death. The existing literature cannot establish whether existing interventions prevent the most serious or lethal forms of SITBs in children and adolescents. Additional research is needed to produce accurate and reliable estimates of treatment effects on these outcomes.

Most outcomes were measured dichotomously (i.e., discrete SITB events), rather than continuously (e.g., SITB severity). It is therefore possible that existing interventions may be more efficacious at reducing SITB severity than preventing SITBs; however, due to an insufficient number of continuous effect sizes, we were unable to directly examine this possibility. Because the majority of examined interventions were designed to target psychopathology broadly defined, rather than SITBs specifically, researchers may have opted to minimize the amount of time spent assessing SITBs by relying on single-item measures. Future studies would benefit from leveraging continuous measures of SITBs to better capture the effects of treatment on SITB severity, rather than simply the presence or absence of SITBs.

Although most psychosocial interventions were described as either “developed” or “adapted” for youth, most were based on existing adult interventions. Rather than modifying preexisting treatments, an alternative approach would be developing novel interventions for youth based on putative causal processes specific to this population. This approach has proved successful in treating other life-threatening behaviors in youth (e.g., family-based treatment for anorexia nervosa^46^). Additional empirical research is warranted to explore this approach for SITBs.

Some may suggest that SITB interventions are less efficacious for youth because SITBs in children and adolescents indicate greater severity. Prior research has demonstrated that SITBs in childhood and adolescence are associated with indicators of severe psychopathology^47^ and poor prognosis^48^, which may lead us to expect a ceiling effect of SITB treatment in youth. However, SITBs in youth may be less entrenched than in adults; thus, interventions implemented at strategic developmental inflection points (i.e., before SITBs become established) may not only reduce the prevalence of SITBs in youth, but also the risk of subsequent psychopathology. Preventing the initial onset of SITBs in youth may be critical for mitigating the burden of SITBs throughout the lifespan^49^.

The ubiquity of medication-only interventions is notable, as no medications have been specifically developed to target SITBs^35^, and medications are rarely formulated specifically for pediatric use^50^. Therefore, dosages may not have been optimized for youth SITB treatment. Concerns related to dosage are not circumscribed to medication; nuances in the implementation of psychosocial interventions (e.g., frequency and length of sessions) can also influence treatment efficacy. Due to an insufficient number of effect sizes in each dosage category, analyzing the effects of dosage was not feasible. Future research is required to quantify the optimal dosage of pharmacological and psychosocial SITB interventions for youth.

Although pooled treatment effects for medication-only interventions were nonsignificant, we detected a statistically significant moderating effect indicating that active arm participants were slightly more likely to experience other/combined SITBs than the control arm. However, medication-only interventions did not appear to increase risk for other SITB categories (including suicide ideation and attempts) and overall effects of medication-only interventions on SITBs did not statistically differ from the effects of either combination or CT/CBT-only interventions. Additional research is needed to determine whether the potential benefits of medication-only interventions outweigh the potential risks^51,52^.

Interventions not yet evaluated with RCTs may be more efficacious than those included in this study, and the next several years will likely result in continued expansion of this literature. Nevertheless, the preponderance of nonsignificant treatment effects and restricted range of variability in our results demonstrate that new interventions must differ meaningfully from existing interventions to produce significant treatment effects. This may require a major paradigm shift in our approach to intervention development and implementation^21,44,53^.

Our primary recommendation for future research is to prioritize the identification of causal mechanisms underlying SITBs with experiments^44,54^, which will facilitate the identification of viable treatment targets. Experiments with SITBs as outcomes are rare, but validated laboratory approximations of SITBs make it possible to safely test causal hypotheses about even severe SITBs. Uncovering SITB causes in youth represents a unique challenge; despite evidence repudiating the misconception that assessing suicidality increases risk^55,56^, guardians may be wary of experiments testing causal hypotheses about SITBs. Additional research examining the safety and validity of translational approaches studying SITBs in children/adolescents may assuage concerns about conducting SITB research in this population.

There is also an urgent need for better dissemination and implementation to address barriers to mental health treatment. Improving access may involve leveraging novel technology^57,58^ and delivering interventions in nontraditional settings^59,60^. Notably, the success of these endeavors requires the identification of SITB causes and the development of more efficacious treatments. Therefore, broad dissemination and implementation are secondary goals which should be addressed further once the causes of SITBs are identified, and more efficacious treatments are developed.

As we work towards improving intervention efficacy, we must consider the current implications of these findings. As noted in this study’s parent meta-analysis^21^, recognizing the limitations of existing interventions is critical. Nevertheless, there may be idiographic treatment effects that were not captured in the current meta-analytic effort. It remains possible that existing interventions may be quite beneficial for some, while producing minimal benefit for most. Given these considerations, it may be useful to prioritize inexpensive, brief, and scalable treatments when possible; they demonstrate comparable efficacy to more expensive, longer, and more intensive treatments.

This meta-analysis indicates that most youth SITB interventions produce nonsignificant treatment effects. Results were largely consistent over time, regardless of intervention type, SITB outcome type, and sample and study characteristics. Although these results are disappointing, we believe that research prioritizing the identification of SITB causes has the potential to produce meaningful reductions in SITBs in child and adolescent populations. We hope that this study catalyzes research prioritizing the identification of SITB causes and exploring novel approaches to the study and treatment of SITBs in youth.

## Supplementary Information

Supplementary Information 1.

Supplementary Figures.

Supplementary Tables.

Supplementary Information 2.

Supplementary Information 3.

## Data Availability

Relevant data are available upon reasonable request to the corresponding author.
