# Supplementary material for: Efficacy of interventions for suicide and self-injury in children and adolescents: a meta-analysis
Source: Sci Rep. 2022 Jul 19;12:12313. doi: 10.1038/s41598-022-16567-8 (PMC9296501; doi:10.1038/s41598-022-16567-8)
Supplement: Supplementary file 1 — Supplementary Information 1. [file 41598_2022_16567_MOESM1_ESM.docx]

**Supplement 1**

1.Alavi, A., Sharifi, B., Ghanizadeh, A. & Dehbozorgi, G. Effectiveness of Cognitive-Behavioral Therapy in Decreasing Suicidal Ideation. Iran J Pediatr 23, 6 (2013).

2.Arango, C. et al. Safety and efficacy of agomelatine in children and adolescents with major depressive disorder receiving psychosocial counselling: a double-blind, randomised, controlled, phase 3 trial in nine countries. The Lancet Psychiatry 9, 113–124 (2022).

3.Asarnow, J. R. et al. Dialectical Behavior Therapy for Suicidal Self-Harming Youth: Emotion Regulation, Mechanisms, and Mediators. Journal of the American Academy of Child & Adolescent Psychiatry 60, 1105-1115.e4 (2021).

4.Asarnow, J. R., Hughes, J. L., Babeva, K. N. & Sugar, C. A. Cognitive-Behavioral Family Treatment for Suicide Attempt Prevention: A Randomized Controlled Trial. Journal of the American Academy of Child & Adolescent Psychiatry 56, 506–514 (2017).

5.Atkinson, S. D. et al. A Double-Blind Efficacy and Safety Study of Duloxetine Flexible Dosing in Children and Adolescents with Major Depressive Disorder. Journal of Child and Adolescent Psychopharmacology 24, 180–189 (2014).

6.Beck, E. et al. Mentalization‐based treatment in groups for adolescents with borderline personality disorder: a randomized controlled trial. J Child Psychol Psychiatr 61, 594–604 (2020).

7.Bonell, C. et al. Effects of the Learning Together intervention on bullying and aggression in English secondary schools (INCLUSIVE): a cluster randomised controlled trial. The Lancet 392, 2452–2464 (2018).

8.Brams, M. et al. SHP465 Mixed Amphetamine Salts in the Treatment of Attention-Deficit/Hyperactivity Disorder in Children and Adolescents: Results of a Randomized, Double-Blind Placebo-Controlled Study. Journal of Child and Adolescent Psychopharmacology 28, 19–28 (2018).

9.Brent, D. A. A Clinical Psychotherapy Trial for Adolescent Depression Comparing Cognitive, Family, and Supportive Therapy. Arch Gen Psychiatry 54, 877 (1997).

10.Brent, D. B. Switching to Another SSRI or to Venlafaxine With or Without Cognitive Behavioral Therapy for Adolescents With SSRI-Resistant Depression:€€The TORDIA Randomized Controlled Trial. 13.

11.Britton, W. B. et al. A randomized controlled pilot trial of classroom-based mindfulness meditation compared to an active control condition in sixth-grade children. Journal of School Psychology 52, 263–278 (2014).

12.Cardo, E. et al. Fast vs. Slow Switching from Stimulants to Atomoxetine in Children and Adolescents with Attention-Deficit/Hyperactivity Disorder. Journal of Child and Adolescent Psychopharmacology 23, 252–261 (2013).

13.Chanen, A. M. et al. Early intervention for adolescents with borderline personality disorder using cognitive analytic therapy: randomised controlled trial. Br J Psychiatry 193, 477–484 (2008).

14.Chavira, D. A. et al. Telephone-assisted, parent-mediated CBT for rural Latino youth with anxiety: A feasibility trial. Cultural Diversity and Ethnic Minority Psychology 24, 429–441 (2018).

15.Childress, A. C., Kollins, S. H., Cutler, A. J., Marraffino, A. & Sikes, C. R. Efficacy, Safety, and Tolerability of an Extended-Release Orally Disintegrating Methylphenidate Tablet in Children 6–12 Years of Age with Attention-Deficit/Hyperactivity Disorder in the Laboratory Classroom Setting. Journal of Child and Adolescent Psychopharmacology 27, 66–74 (2017).

16.Cotgrove, A., Zirinsky, L., Black, D. & Weston, D. Secondary prevention of attempted suicide in adolescence. Journal of Adolescence 18, 569–577 (1995).

17.Cottrell, D. J. et al. Effectiveness of systemic family therapy versus treatment as usual for young people after self-harm: a pragmatic, phase 3, multicentre, randomised controlled trial. The Lancet Psychiatry 5, 203–216 (2018).

18.Czyz, E. K., King, C. A. & Biermann, B. J. Motivational Interviewing-Enhanced Safety Planning for Adolescents at High Suicide Risk: A Pilot Randomized Controlled Trial. Journal of Clinical Child & Adolescent Psychology 48, 250–262 (2019).

19.Detke, H. C., DelBello, M. P., Landry, J. & Usher, R. W. Olanzapine/Fluoxetine Combination in Children and Adolescents With Bipolar I Depression: A Randomized, Double-Blind, Placebo-Controlled Trial. Journal of the American Academy of Child & Adolescent Psychiatry 54, 217–224 (2015).

20.Diamond, G. S. et al. Attachment-Based Family Therapy for Adolescents with Suicidal Ideation: A Randomized Controlled Trial. ADOLESCENT PSYCHIATRY 49, 10 (2010).

21.Dobias, M. L., Schleider, J. L., Jans, L. & Fox, K. R. An online, single-session intervention for adolescent self-injurious thoughts and behaviors: Results from a randomized trial. Behaviour Research and Therapy 147, 103983 (2021).

22.Donaldson, D., Spirito, A. & Esposito-Smythers, C. Treatment for Adolescents Following a Suicide Attempt: Results of a Pilot Trial. Journal of the American Academy of Child & Adolescent Psychiatry 44, 113–120 (2005).

23.Durgam, S. et al. A Phase 3, Double-Blind, Randomized, Placebo-Controlled Study of Vilazodone in Adolescents with Major Depressive Disorder. Pediatr Drugs 20, 353–363 (2018).

24.Emslie, G. J. et al. Fluoxetine Versus Placebo in Preventing Relapse of Major Depression in Children and Adolescents. Am J Psychiatry 9 (2008).

25.Emslie, G. J., Findling, R. L., Yeung, P. P., Kunz, N. R. & Li, Y. Venlafaxine ER for the Treatment of Pediatric Subjects With Depression. Journal of the American Academy of Child & Adolescent Psychiatry 46, 479–488 (2007).

26.Emslie, G. J. et al. Fluoxetine for Acute Treatment of Depression in Children and Adolescents: A Placebo-Controlled, Randomized Clinical Trial. Journal of the American Academy of Child & Adolescent Psychiatry 41, 1205–1215 (2002).

27.Emslie, G. J. et al. A Double-Blind Efficacy and Safety Study of Duloxetine Fixed Doses in Children and Adolescents with Major Depressive Disorder. Journal of Child and Adolescent Psychopharmacology 24, 170–179 (2014).

28.Emslie, G. J., Ventura, D., Korotzer, A. & Tourkodimitris, S. Escitalopram in the Treatment of Adolescent Depression: A Randomized Placebo-Controlled Multisite Trial. Journal of the American Academy of Child & Adolescent Psychiatry 48, 721–729 (2009).

29.Emslie, G. J. et al. Paroxetine Treatment in Children and Adolescents With Major Depressive Disorder: A Randomized, Multicenter, Double-Blind, Placebo-Controlled Trial. Journal of the American Academy of Child & Adolescent Psychiatry 45, 709–719 (2006).

30.Emslie, G. et al. Treatment for Adolescents With Depression Study (TADS). Journal of the American Academy of Child & Adolescent Psychiatry 45, 1440–1455 (2006).

31.Esposito-Smythers, C., Hadley, W., Curby, T. W. & Brown, L. K. Randomized pilot trial of a cognitive-behavioral alcohol, self-harm, and HIV prevention program for teens in mental health treatment. Behaviour Research and Therapy 89, 49–56 (2017).

32.Esposito-Smythers, C., Spirito, A., Kahler, C. W., Hunt, J. & Monti, P. Treatment of co-occurring substance abuse and suicidality among adolescents: A randomized trial. Journal of Consulting and Clinical Psychology 79, 728–739 (2011).

33.Findling, R. L., Pathak, S., Earley, W. R., Liu, S. & DelBello, M. P. Efficacy and Safety of Extended-Release Quetiapine Fumarate in Youth with Bipolar Depression: An 8 Week, Double-Blind, Placebo-Controlled Trial. Journal of Child and Adolescent Psychopharmacology 24, 325–335 (2014).

34.Findling, R. L. et al. Ziprasidone in Adolescents with Schizophrenia: Results from a Placebo-Controlled Efficacy and Long-Term Open-Extension Study. Journal of Child and Adolescent Psychopharmacology 23, 531–544 (2013).

35.Findling, R. L. et al. Adjunctive Maintenance Lamotrigine for Pediatric Bipolar I Disorder: A Placebo-Controlled, Randomized Withdrawal Study. Journal of the American Academy of Child & Adolescent Psychiatry 54, 1020-1031.e3 (2015).

36.Findling, R. L. et al. Asenapine for the Acute Treatment of Pediatric Manic or Mixed Episode of Bipolar I Disorder. Journal of the American Academy of Child & Adolescent Psychiatry 54, 1032–1041 (2015).

37.Findling, R. L. et al. Safety and Efficacy from an 8 Week Double-Blind Trial and a 26 Week Open-Label Extension of Asenapine in Adolescents with Schizophrenia. Journal of Child and Adolescent Psychopharmacology 25, 384–396 (2015).

38.Findling, R. L., McKenna, K., Earley, W. R., Stankowski, J. & Pathak, S. Efficacy and Safety of Quetiapine in Adolescents with Schizophrenia Investigated in a 6-Week, Double-Blind, Placebo-Controlled Trial. Journal of Child and Adolescent Psychopharmacology 22, 327–342 (2012).

39.Findling, R. L., Robb, A. & Bose, A. Escitalopram in the Treatment of Adolescent Depression: A Randomized, Double-Blind, Placebo-Controlled Extension Trial. Journal of Child and Adolescent Psychopharmacology 23, 468–480 (2013).

40.Geller, B. A Randomized Controlled Trial of Risperidone, Lithium, or Divalproex Sodium for Initial Treatment of Bipolar I Disorder, Manic or Mixed Phase, in Children and Adolescents. Arch Gen Psychiatry 69, 515 (2012).

41.Geller, D. A. et al. Paroxetine Treatment in Children and Adolescents With Obsessive-Compulsive Disorder: A Randomized, Multicenter, Double-Blind, Placebo-Controlled Trial. Journal of the American Academy of Child & Adolescent Psychiatry 43, 1387–1396 (2004).

42.Gibbons, R. D., Brown, C. H., Hur, K., Davis, J. M. & Mann, J. J. Suicidal Thoughts and Behavior With Antidepressant Treatment: Reanalysis of the Randomized Placebo-Controlled Studies of Fluoxetine and Venlafaxine. Arch Gen Psychiatry 69, 580 (2012).

43.Goldstein, T. R. et al. Dialectical Behavior Therapy for Adolescents with Bipolar Disorder: Results from a Pilot Randomized Trial. Journal of Child and Adolescent Psychopharmacology 25, 140–149 (2015).

44.Goodyer, I. et al. Selective serotonin reuptake inhibitors (SSRIs) and routine specialist care with and without cognitive behaviour therapy in adolescents with major depression: randomised controlled trial. BMJ 335, 142 (2007).

45.Griffiths, H. et al. Efficacy of Mentalization-based group therapy for adolescents: the results of a pilot randomised controlled trial. BMC Psychiatry 19, 167 (2019).

46.Grupp-Phelan, J. et al. Effect of a Motivational Interviewing–Based Intervention on Initiation of Mental Health Treatment and Mental Health After an Emergency Department Visit Among Suicidal Adolescents: A Randomized Clinical Trial. JAMA Netw Open 2, e1917941 (2019).

47.Hammad, T. A., Laughren, T. & Racoosin, J. Suicidality in Pediatric Patients Treated With Antidepressant Drugs. ARCH GEN PSYCHIATRY 63, 8 (2006).

48.Hazell, P. L. et al. Group Therapy for Repeated Deliberate Self-Harm in Adolescents: Failure of Replication of a Randomized Trial. Journal of the American Academy of Child & Adolescent Psychiatry 48, 662–670 (2009).

49.Hengartner, M. P. & Plöderl, M. Suicidality and other severe psychiatric events with duloxetine: Re-analysis of safety data from a placebo-controlled trial for juvenile fibromyalgia. JRS 32, 209–218 (2021).

50.Herpertz-Dahlmann, B. et al. Day-patient treatment after short inpatient care versus continued inpatient treatment in adolescents with anorexia nervosa (ANDI): a multicentre, randomised, open-label, non-inferiority trial. The Lancet 383, 1222–1229 (2014).

51.Hervas, A. et al. Efficacy and safety of extended-release guanfacine hydrochloride in children and adolescents with attention-deficit/hyperactivity disorder: A randomized, controlled, Phase III trial. European Neuropsychopharmacology 24, 1861–1872 (2014).

52.Högberg, G. & Hällström, T. Mood Regulation Focused CBT Based on Memory Reconsolidation, Reduced Suicidal Ideation and Depression in Youth in a Randomised Controlled Study. IJERPH 15, 921 (2018).

53.Huey, S. J. et al. Multisystemic Therapy Effects on Attempted Suicide by Youths Presenting Psychiatric Emergencies. Journal of the American Academy of Child & Adolescent Psychiatry 43, 183–190 (2004).

54.Ichikawa, H. et al. Aripiprazole in the Treatment of Irritability in Children and Adolescents with Autism Spectrum Disorder in Japan: A Randomized, Double-blind, Placebo-controlled Study. Child Psychiatry Hum Dev 48, 796–806 (2017).

55.Johnson, J. K. et al. A Phase II Double-Blind, Placebo-Controlled, Efficacy and Safety Study of SPN-812 (Extended-Release Viloxazine) in Children With ADHD. J Atten Disord 24, 348–358 (2020).

56.Kaess, M. et al. Effectiveness of a brief psychotherapeutic intervention compared with treatment as usual for adolescent nonsuicidal self-injury: a single-centre, randomised controlled trial. Eur Child Adolesc Psychiatry 29, 881–891 (2020).

57.Kaminer, Y., Burleson, J. A., Goldston, D. B. & Burke, R. H. Suicidal Ideation among Adolescents with Alcohol Use Disorders during Treatment and Aftercare. Am J Addict 15, 43–49 (2006).

58.King, C. A. et al. LET’s CONNECT Community Mentorship Program for Adolescents with Peer Social Problems: A Randomized Intervention Trial. American J of Comm Psychol 68, 310–322 (2021).

59.King, C. A. et al. The Youth-Nominated Support Team–Version II for suicidal adolescents: A randomized controlled intervention trial. Journal of Consulting and Clinical Psychology 77, 880–893 (2009).

60.King, C. A. et al. Youth-Nominated Support Team for suicidal adolescents (Version 1): A randomized controlled trial. Journal of Consulting and Clinical Psychology 74, 199–206 (2006).

61.Klim-Conforti, P. et al. The Impact of a Harry Potter-Based Cognitive-Behavioral Therapy Skills Curriculum on Suicidality and Well-being in Middle Schoolers: A Randomized Controlled Trial. Journal of Affective Disorders 286, 134–141 (2021).

62.Kolko, D. J., Lindhiem, O., Hart, J. & Bukstein, O. G. Evaluation of a Booster Intervention Three Years After Acute Treatment for Early-Onset Disruptive Behavior Disorders. J Abnorm Child Psychol 42, 383–398 (2014).

63.Kollins, S. H. et al. A Randomized, Controlled Laboratory Classroom Study of Serdexmethylphenidate and d-Methylphenidate Capsules in Children with Attention-Deficit/Hyperactivity Disorder. Journal of Child and Adolescent Psychopharmacology 31, 597–609 (2021).

64.Kryzhanovskaya, L. A., Xu, W. & Dittmann, R. W. The Safety of Olanzapine in Adolescents With Schizophrenia or Bipolar I Disorder: A Pooled Analysis of 4 Clinical Trials. J Clin Psychiatry 12 (2009).

65.le Grange, D., Crosby, R. D., Rathouz, P. J. & Leventhal, B. L. A Randomized Controlled Comparison of Family-Based Treatment and Supportive Psychotherapy for Adolescent Bulimia Nervosa. Arch Gen Psychiatry 64, 1049 (2007).

66.Le Noury, J. et al. Study 329 continuation phase: Safety and efficacy of paroxetine and imipramine in extended treatment of adolescent major depression. JRS 28, 143–161 (2016).

67.Listug-Lunde, L. B. A Cognitive-Behavioral Treatment for Depression in Native American Middle-School Students. American Indian and Alaskman Native Mental Health Research 20, 16–34 (2013).

68.March, J. S., Entusah, A. R., Rynn, M., Albano, A. M. & Tourian, K. A. A Randomized Controlled Trial of Venlafaxine ER Versus Placebo in Pediatric Social Anxiety Disorder. Biological Psychiatry 62, 1149–1154 (2007).

69.McCarty, C. A. et al. Effect of Collaborative Care on Persistent Postconcussive Symptoms in Adolescents: A Randomized Clinical Trial. JAMA Netw Open 4, e210207 (2021).

70.McGough, J. J. et al. Double-Blind, Sham-Controlled, Pilot Study of Trigeminal Nerve Stimulation for Attention-Deficit/Hyperactivity Disorder. Journal of the American Academy of Child & Adolescent Psychiatry 58, 403-411.e3 (2019).

71.McManama O’Brien, K. H. et al. Feasibility, acceptability, and preliminary effects of a brief alcohol intervention for suicidal adolescents in inpatient psychiatric treatment. Journal of Substance Abuse Treatment 94, 105–112 (2018).

72.Mehlum, L. et al. Dialectical Behavior Therapy Compared With Enhanced Usual Care for Adolescents With Repeated Suicidal and Self-Harming Behavior: Outcomes Over a One-Year Follow-Up. Journal of the American Academy of Child & Adolescent Psychiatry 55, 295–300 (2016).

73.Mehlum, L. et al. Dialectical Behavior Therapy for Adolescents With Repeated Suicidal and Self-harming Behavior: A Randomized Trial. Journal of the American Academy of Child & Adolescent Psychiatry 53, 1082–1091 (2014).

74.Melvin, G. A. et al. A Comparison of Cognitive-Behavioral Therapy, Sertraline, and Their Combination for Adolescent Depression. Journal of the American Academy of Child & Adolescent Psychiatry 45, 1151–1161 (2006).

75.Merry, S. N. et al. The effectiveness of SPARX, a computerised self help intervention for adolescents seeking help for depression: randomised controlled non-inferiority trial. BMJ 344, e2598–e2598 (2012).

76.Miklowitz, D. J. et al. Effects of family-focused therapy on suicidal ideation and behavior in youth at high risk for bipolar disorder. Journal of Affective Disorders 275, 14–22 (2020).

77.Nasser, A. et al. A Phase III, Randomized, Placebo-controlled Trial to Assess the Efficacy and Safety of Once-daily SPN-812 (Viloxazine Extended-release) in the Treatment of Attention-deficit/Hyperactivity Disorder in School-age Children. Clinical Therapeutics 42, 1452–1466 (2020).

78.Nordh, M. et al. Therapist-Guided Internet-Delivered Cognitive Behavioral Therapy vs Internet-Delivered Supportive Therapy for Children and Adolescents With Social Anxiety Disorder: A Randomized Clinical Trial. JAMA Psychiatry 78, 705 (2021).

79.Owen, R. et al. Aripiprazole in the Treatment of Irritability in Children and Adolescents With Autistic Disorder. PEDIATRICS 124, 1533–1540 (2009).

80.Peltonen, K. & Kangaslampi, S. Treating children and adolescents with multiple traumas: a randomized clinical trial of narrative exposure therapy. European Journal of Psychotraumatology 10, 1558708 (2019).

81.Perepletchikova, F. et al. Randomized Clinical Trial of Dialectical Behavior Therapy for Preadolescent Children With Disruptive Mood Dysregulation Disorder: Feasibility and Outcomes. Journal of the American Academy of Child & Adolescent Psychiatry 56, 832–840 (2017).

82.Perry, Y. et al. Effects of a classroom-based educational resource on adolescent mental health literacy: A cluster randomised controlled trial. Journal of Adolescence 37, 1143–1151 (2014).

83.Pineda, J. & Dadds, M. R. Family Intervention for Adolescents With Suicidal Behavior: A Randomized Controlled Trial and Mediation Analysis. Journal of the American Academy of Child & Adolescent Psychiatry 52, 851–862 (2013).

84.Prout, T. A. et al. Randomized controlled trial of Regulation Focused Psychotherapy for children: A manualized psychodynamic treatment for externalizing behaviors. Psychotherapy Research 32, 555–570 (2022).

85.Reid, A. M. et al. Side-effects of SSRIs disrupt multimodal treatment for pediatric OCD in a randomized-controlled trial. Journal of Psychiatric Research 71, 140–147 (2015).

86.Robb, A. S. et al. Efficacy, Safety, and Tolerability of a Novel Methylphenidate Extended-Release Oral Suspension (MEROS) in ADHD. J Atten Disord 21, 1180–1191 (2017).

87.Rossouw, T. I. & Fonagy, P. Mentalization-Based Treatment for Self-Harm in Adolescents: A Randomized Controlled Trial. Journal of the American Academy of Child & Adolescent Psychiatry 51, 1304-1313.e3 (2012).

88.Saito, T. et al. Using the drug repositioning approach to develop a novel therapy, tipepidine hibenzate sustained-release tablet (TS-141), for children and adolescents with attention-deficit/hyperactivity disorder. BMC Psychiatry 20, 530 (2020).

89.Sallee, F. et al. Randomized, Double-Blind, Placebo-Controlled Trial Demonstrates the Efficacy and Safety of Oral Aripiprazole for the Treatment of Tourette’s Disorder in Children and Adolescents. Journal of Child and Adolescent Psychopharmacology 27, 771–781 (2017).

90.Sangal, R. B., Blumer, J. L., Lankford, D. A., Grinnell, T. A. & Huang, H. Eszopiclone for Insomnia Associated With Attention-Deficit/Hyperactivity Disorder. PEDIATRICS 134, e1095–e1103 (2014).

91.Santamarina‐Perez, P. et al. Adapted Dialectical Behavior Therapy for Adolescents with a High Risk of Suicide in a Community Clinic: A Pragmatic Randomized Controlled Trial. Suicide Life Threat Behav 50, 652–667 (2020).

92.Schilling, E. A., Aseltine, R. H. & James, A. The SOS Suicide Prevention Program: Further Evidence of Efficacy and Effectiveness. Prev Sci 17, 157–166 (2016).

93.Schiltz, H. K. et al. Changes in Depressive Symptoms Among Adolescents with ASD Completing the PEERS® Social Skills Intervention. J Autism Dev Disord 48, 834–843 (2018).

94.Singh, J., Robb, A., Vijapurkar, U., Nuamah, I. & Hough, D. A Randomized, Double-Blind Study of Paliperidone Extended-Release in Treatment of Acute Schizophrenia in Adolescents. Biological Psychiatry 70, 1179–1187 (2011).

95.Spirito, A. et al. Concurrent Treatment for Adolescent and Parent Depressed Mood and Suicidality: Feasibility, Acceptability, and Preliminary Findings. Journal of Child and Adolescent Psychopharmacology 25, 131–139 (2015).

96.Strawn, J. R. et al. Extended Release Guanfacine in Pediatric Anxiety Disorders: A Pilot, Randomized, Placebo-Controlled Trial. Journal of Child and Adolescent Psychopharmacology 27, 29–37 (2017).

97.Strawn, J. R. et al. A Randomized, Placebo-Controlled Study of Duloxetine for the Treatment of Children and Adolescents With Generalized Anxiety Disorder. Journal of the American Academy of Child & Adolescent Psychiatry 54, 283–293 (2015).

98.TADS team. Fluoxetine, Cognitive-Behavioral Therapy, and Their Combination for Adolescents With Depression: Treatment for Adolescents With Depression Study (TADS) Randomized Controlled Trial. JAMA 292, 807 (2004).

99.TADS team. The Treatment for Adolescents With Depression Study (TADS): Long-term Effectiveness and Safety Outcomes. Arch Gen Psychiatry 64, 1132 (2007).

100.Tang, T.-C., Jou, S.-H., Ko, C.-H., Huang, S.-Y. & Yen, C.-F. Randomized study of school-based intensive interpersonal psychotherapy for depressed adolescents with suicidal risk and parasuicide behaviors. Psychiatry and Clinical Neurosciences 63, 463–470 (2009).

101.Tingey, L. et al. Behavioral and Mental Health outcomes from an RCT of a Youth Entrepreneurship Intervention among Native American Adolescents. Children and Youth Services Review 119, 105603 (2020).

102.Tompson, M. C., Langer, D. A. & Asarnow, J. R. Development and efficacy of a family-focused treatment for depression in childhood. Journal of Affective Disorders 276, 686–695 (2020).

103.von Knorring, A.-L., Olsson, G. I., Thomsen, P. H., Lemming, O. M. & Hultén, A. A Randomized, Double-blind, Placebo-controlled Study of Citalopram in Adolescents With Major Depressive Disorder. Journal of Clinical Psychopharmacology 26, 311–315 (2006).

104.Wagner, K. D. Efficacy of Sertraline in the Treatment of Children and Adolescents With Major Depressive DisorderTwo Randomized Controlled Trials. JAMA 290, 1033 (2003).

105.Wagner, K. D. et al. A Multicenter, Randomized, Double-blind, Placebo-Controlled Trial ofParoxetine in Children and Adolescents With Social Anxiety Disorder. Arch Gen Psychiatry 61, 1153 (2004).

106.Wagner, K. D., Jonas, J., Findling, R. L., Ventura, D. & Saikali, K. A Double-Blind, Randomized, Placebo-Controlled Trial of Escitalopram in the Treatment of Pediatric Depression. Journal of the American Academy of Child & Adolescent Psychiatry 45, 280–288 (2006).

107.Walkup, J. T. et al. Cognitive Behavioral Therapy, Sertraline, or a Combination in Childhood Anxiety. N Engl J Med 359, 2753–2766 (2008).

108.Weihs, K. L. et al. Desvenlafaxine Versus Placebo in a Fluoxetine-Referenced Study of Children and Adolescents with Major Depressive Disorder. Journal of Child and Adolescent Psychopharmacology 28, 36–46 (2018).

109.Weinstein, S. M., Cruz, R. A., Isaia, A. R., Peters, A. T. & West, A. E. Child- and Family-Focused Cognitive Behavioral Therapy for Pediatric Bipolar Disorder: Applications for Suicide Prevention. Suicide Life Threat Behav 48, 797–811 (2018).

110.Weiss, M. D., Cutler, A. J., Kollins, S. H. & Donnelly, G. A. E. Efficacy and Safety of a Long-Acting Multilayer-Release Methylphenidate Formulation (PRC-063) in the Treatment of Adolescent Attention-Deficit/Hyperactivity Disorder: A Randomized, Double-Blind Clinical Trial with a 6-Month Open-Label Extension. Journal of Child and Adolescent Psychopharmacology 31, 610–622 (2021).

111.Wigal, S. B., Childress, A. C., Belden, H. W. & Berry, S. A. NWP06, an Extended-Release Oral Suspension of Methylphenidate, Improved Attention-Deficit/Hyperactivity Disorder Symptoms Compared with Placebo in a Laboratory Classroom Study. Journal of Child and Adolescent Psychopharmacology 23, 3–10 (2013).

112.Wyman, P. A. et al. An Outcome Evaluation of the Sources of Strength Suicide Prevention Program Delivered by Adolescent Peer Leaders in High Schools. Am J Public Health 100, 1653–1661 (2010).
