# Supplementary material for: Efficacy of interventions for suicide and self-injury in children and adolescents: a meta-analysis
Source: Sci Rep. 2022 Jul 19;12:12313. doi: 10.1038/s41598-022-16567-8 (PMC9296501; doi:10.1038/s41598-022-16567-8)

**Supplement 2**

**Figure S1.** Funnel Plot for Overall Effects on Binary Outcomes


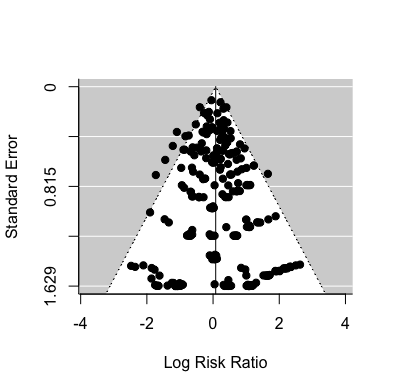


**Figure S2.** Funnel Plot for Overall Effects on Continuous Outcomes

**
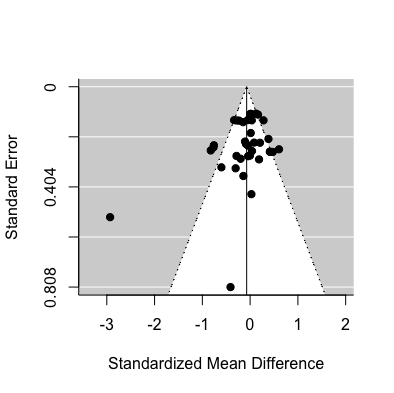
**

**Figure S3.** Funnel Plot for Overall Effects on Suicide Ideation Binary Outcomes

**
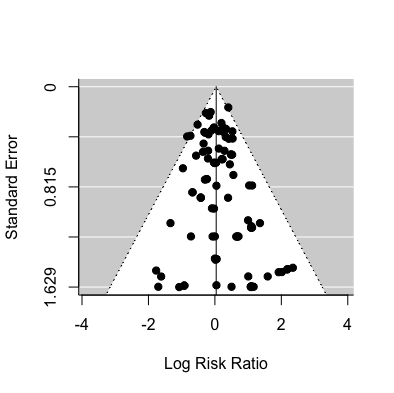
**

**Figure S4.** Funnel Plot for Overall Effects on Suicide Ideation Continuous Outcomes


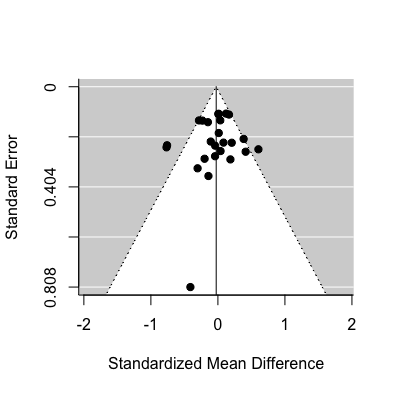


**Figure S5.** Funnel Plot for Overall Effects on Suicide Attempt Binary Outcomes


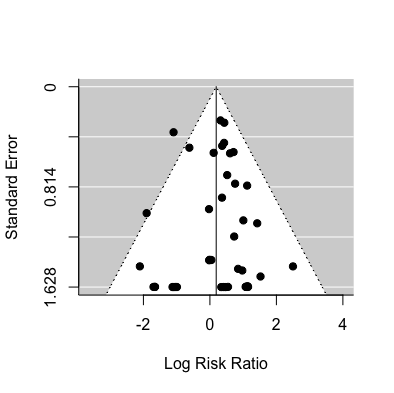


**Figure S6.** Funnel Plot for Overall Effects on Suicide Death Binary Outcomes


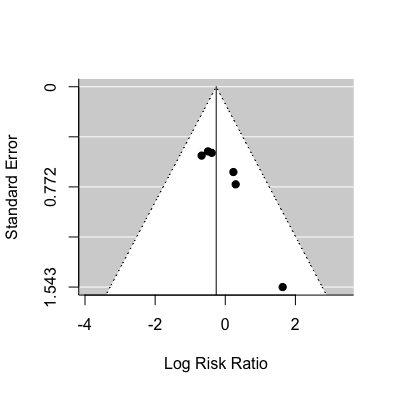


**Figure S7.** Funnel Plot for Overall Effects on NSSI Binary Outcomes


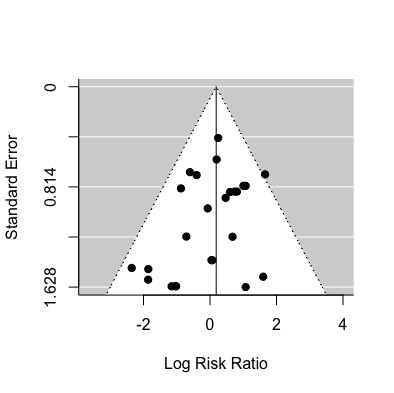


**Figure S8.** Funnel Plot for Overall Effects on Self-Harm Binary Outcomes


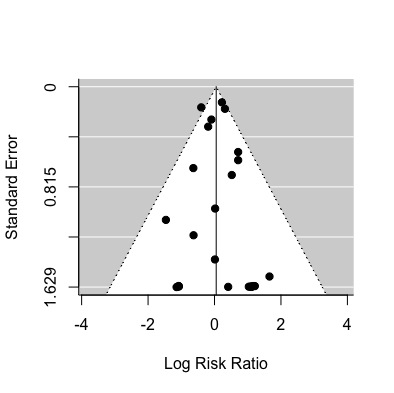


**Figure S9.** Funnel Plot for Overall Effects on Self-Harm Continuous Outcomes

**
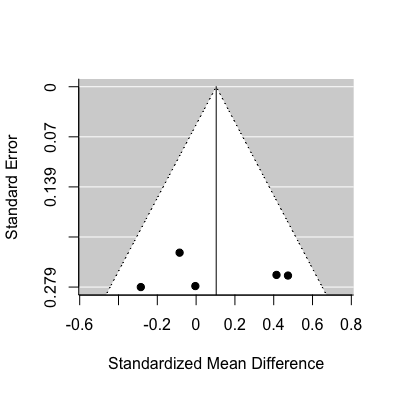
**

**Figure S10.** Funnel Plot for Overall Effects on Other/Combined SITB Binary Outcomes


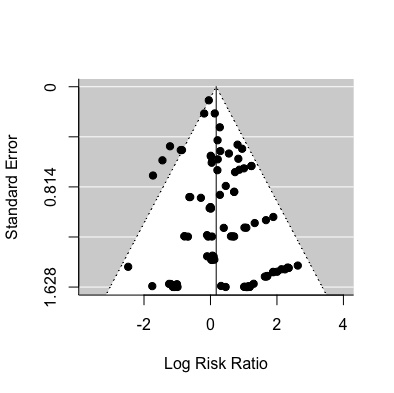

Supplement: Supplementary file 2 — Supplementary Figures. [file 41598_2022_16567_MOESM2_ESM.docx]
