# Supplementary material for: Efficacy of interventions for suicide and self-injury in children and adolescents: a meta-analysis
Source: Sci Rep. 2022 Jul 19;12:12313. doi: 10.1038/s41598-022-16567-8 (PMC9296501; doi:10.1038/s41598-022-16567-8)
Supplement: Supplementary file 3 — Supplementary Tables. [file 41598_2022_16567_MOESM3_ESM.docx]

**Supplement 3**

Table S1. Moderator analyses for Suicide Ideation.

|  |  | **Overall** | | | | | |
| --- | --- | --- | --- | --- | --- | --- | --- |
|  | **Binary** | | |  |  | **Continuous** | |
|  | **n** | | **RR [95% CI]** |  |  | **n** | ***g* [95% CI]** |
| **Pooled Effects** |  | |  |  |  |  |  |
| **Specific Intervention Type** |  | |  |  |  |  |  |
| Medication Only | 105 | | 1.04 [0.90, 1.19] |  |  | 0 | - |
| Psychotherapy and Medication Combined | 11 | | 0.99 [0.68, 1.43] |  |  | 8 | -0.06 [-0.17, 0.04] |
| CT/CBT | 4 | | - |  |  | 8 | -0.12 [-0.29, 0.05] |
| Mixed Psychotherapy Modalities | 0 | | - |  |  | 5 | 0.13 [-0.07, 0.33] |
| Psychoeducation | 2 | | - |  |  | 4 | - |
| DBT | 1 | | - |  |  | 1 | - |
| Parenting Skills Training | 2 | | - |  |  | 0 | - |
|  |  | |  |  |  |  |  |
| **Control Group Type** |  | |  |  |  |  |  |
| No Treatment | 3 | | - |  |  | 4 | - |
| Placebo | 65 | | 0.99 [0.83, 1.19] |  |  | 2 | - |
| Active Treatment | 58 | | 1.11 [0.96, 1.29] |  |  | 27 | -0.0003 [-0.09, 0.09] |
|  |  | |  |  |  |  |  |
| **Medication Class** |  | |  |  |  |  |  |
| Selective Serotonin Reuptake Inhibitor | 20 | | 1.11 [0.81, 1.52] |  |  | 1 | - |
| Atypical Antipsychotic | 26 | | 1.12 [0.82, 1.53] |  |  | 0 | - |
| Selective Norepinephrine Reuptake Inhibitor | 24 | | 1.15 [0.91, 1.45] |  |  | 0 | - |
| Alpha-2 Adrenergic Agonist | 8 | | 0.98 [0.52, 1.85] |  |  | 0 | - |
| Hypnotic | 5 | | 0.64 [0.27, 1.50] |  |  | 0 | - |
| Atypical Antidepressant | 12 | | 0.78 [0.39, 1.59] |  |  | 0 | - |
| Mood Stabilizer | 2 | | - |  |  | 0 | - |
|  |  | |  |  |  |  |  |
| **Sample Severity** |  | |  |  |  |  |  |
| General | 16 | | 0.86 [0.69, 1.06] |  |  | 6 | -0.18 [-0.43, 0.06] |
| Clinical | 110 | | 1.08 [.95, 1.22] |  |  | 16 | -0.03 [-0.11, 0.06] |
| SITB | 0 | | - |  |  | 11 | 0.04 [-0.14, 0.22] |
|  |  | |  |  |  |  |  |
| **Age Group** |  | |  |  |  |  |  |
| Children | 1 | | - |  |  | 0 | - |
| Adolescents | 112 | | 1.00 [0.89, 1.11] |  |  | 31 | -0.03 [-0.13, 0.06] |
|  |  | |  |  |  |  |  |
| **Intervention Target Type** |  | |  |  |  |  |  |
| SITBs | 11 | | 0.96 [0.80, 1.15] |  |  | 17 | -0.04 [-0.20, 0.13] |
| Psychopathology | 108 | | 1.06 [0.92, 1.21] |  |  | 14 | -0.02 [-0.11, 0.07] |
| Other | 7 | | 0.84 [0.28, 2.49] |  |  | 2 | - |
|  |  | |  |  |  |  |  |
| **Treatment Components** |  | |  |  |  |  |  |
| Individual Only | 112 | | 1.06 [0.93, 1.21] |  |  | 10 | 0.02 [-0.14, 0.18] |
| Family Only | 2 | | - |  |  | 1 | - |
| Group Only | 2 | | - |  |  | 2 | - |
| Individual and Family | 8 | | 0.99 [0.64, 1.53] |  |  | 14 | -0.04 [-0.13, 0.05] |
| Individual, Family, and Group | 0 | | - |  |  | 3 | - |
| School-based | 2 | | - |  |  | 3 | - |
| Individual Skills Training Provided | 112 | | 1.06 [0.93, 1.21] |  |  | 10 | 0.02 [-0.14, 0.18] |
| Designed or Adapted for Adolescents | 18 | | 0.96 [0.78, 1.19] |  |  | 30 | -0.04 [-0.15, 0.06] |
|  |  | |  |  |  |  |  |
| **Study Quality** |  | |  |  |  |  |  |
| Weak | 43 | | 1.02 [0.88, 1.19] |  |  | 19 | -0.02 [-0.15, 0.10] |
| Moderate | 79 | | 0.99 [0.83, 1.18] |  |  | 11 | -0.02 [-0.13, 0.09] |
| Strong | 4 | | - |  |  | 3 | - |
|  |  | |  |  |  |  |  |
| **Therapist Training and Adherence** |  | |  |  |  |  |  |
| Therapist Adherence Check | 91 | | 1.16 [0.91, 1.48] |  |  | 23 | -0.03 [-0.15, 0.08] |
| Therapist Pre-Treatment Training | 86 | | 1.16 [0.91, 1.47] |  |  | 25 | -0.03 [-0.14, 0.0] |

*Note.* Estimates were not reported for analyses involving fewer than five effect sizes to improve the reliability and accuracy of estimates. n = number of effect sizes, RR = weighted mean risk ratio, 95% CI = 95% confidence interval. Dashes indicate unavailable information. Bold indicates an effect estimate which is significantly different from pooled effects (i.e., nonoverlapping confidence intervals).

Table S2. Moderator analyses for Suicide Attempt.

|  |  | **Overall** | | | | | |
| --- | --- | --- | --- | --- | --- | --- | --- |
|  | **Binary** | | |  |  | **Continuous** | |
|  | **n** | | **RR [95% CI]** |  |  | **n** | ***g* [95% CI]** |
| **Pooled Effects** |  | |  |  |  |  |  |
| **Specific Intervention Type** |  | |  |  |  |  |  |
| Medication Only | 26 | | 1.60 [0.94, 2.73] |  |  | 2 | - |
| Psychotherapy and Medication Combined | 9 | | 1.14 [0.70, 1.86] |  |  | 0 | - |
| CT/CBT | 9 | | 1.46 [0.84, 2.54] |  |  | 1 | - |
| Mixed Psychotherapy Modalities | 0 | | - |  |  | 0 | - |
| Psychoeducation | 2 | | - |  |  | 0 | - |
| DBT | 0 | | - |  |  | 0 | - |
| Parenting Skills Training | 2 | | - |  |  | 0 | - |
|  |  | |  |  |  |  |  |
| **Control Group Type** |  | |  |  |  |  |  |
| No Treatment | 1 | | - |  |  | 1 | - |
| Placebo | 21 | | 1.41 [0.78, 2.54] |  |  | 1 | - |
| Active Treatment | 31 | | 1.33 [1.05, 1.68] |  |  | 1 | - |
|  |  | |  |  |  |  |  |
| **Medication Class** |  | |  |  |  |  |  |
| Selective Serotonin Reuptake Inhibitor | 12 | | 1.20 [0.65, 2.22] |  |  | 2 | - |
| Atypical Antipsychotic | 4 | | - |  |  | 0 | - |
| Selective Norepinephrine Reuptake Inhibitor | 5 | | 0.45 [0.11, 1.79] |  |  | 0 | - |
| Alpha-2 Adrenergic Agonist | 0 | | - |  |  | 0 | - |
| Hypnotic | 0 | | - |  |  | 0 | - |
| Atypical Antidepressant | 3 | | - |  |  | 0 | - |
| Mood Stabilizer | 1 | | - |  |  | 0 | - |
|  |  | |  |  |  |  |  |
| **Sample Severity** |  | |  |  |  |  |  |
| General | 4 | | - |  |  | 1 | - |
| Clinical | 44 | | 1.41 [1.03, 1.92] |  |  | 2 | - |
| SITB | 5 | | 1.31 [0.80, 2.16] |  |  | 0 | - |
|  |  | |  |  |  |  |  |
| **Age Group** |  | |  |  |  |  |  |
| Children | 0 | | - |  |  | 0 | - |
| Adolescents | 52 | | 1.21 [0.94, 1.55] |  |  | 3 | - |
|  |  | |  |  |  |  |  |
| **Intervention Target Type** |  | |  |  |  |  |  |
| SITBs | 13 | | 0.87 [0.52, 1.46] |  |  | 1 | - |
| Psychopathology | 38 | | 1.49 [1.08, 2.06] |  |  | 2 | - |
| Other | 2 | | - |  |  | 0 | - |
|  |  | |  |  |  |  |  |
| **Treatment Components** |  | |  |  |  |  |  |
| Individual Only | 36 | | 1.47 [1.05, 2.06] |  |  | 2 | - |
| Family Only | 2 | | - |  |  | 0 | - |
| Group Only | 1 | | - |  |  | 0 | - |
| Individual and Family | 13 | | 1.25 [0.80, 1.95] |  |  | 0 | - |
| Individual, Family, and Group | 0 | | - |  |  | 0 | - |
| School-based | 1 | | - |  |  | 0 | - |
| Individual Skills Training Provided | 22 | | 1.15 [0.83, 1.59] |  |  | 1 | - |
| Designed or Adapted for Adolescents | 22 | | 1.16 [0.82, 1.64] |  |  | 1 | - |
|  |  | |  |  |  |  |  |
| **Study Quality** |  | |  |  |  |  |  |
| Weak | 25 | | 1.01 [0.70, 1.44] |  |  | 1 | - |
| Moderate | 25 | | 1.37 [0.96, 1.96] |  |  | 2 | - |
| Strong |  | |  |  |  |  |  |
|  |  | |  |  |  |  |  |
| **Therapist Training and Adherence** |  | |  |  |  |  |  |
| Therapist Adherence Check | 18 | | 1.25 [0.90, 1.74] |  |  | 0 | - |
| Therapist Pre-Treatment Training | 17 | | 1.29 [0.92, 1.80] |  |  | 0 | - |

*Note.* Estimates were not reported for analyses involving fewer than five effect sizes to improve the reliability and accuracy of estimates. n = number of effect sizes, RR = weighted mean risk ratio, 95% CI = 95% confidence interval. Dashes indicate unavailable information. Bold indicates an effect estimate which is significantly different from pooled effects (i.e., nonoverlapping confidence intervals).

Table S3. Moderator analyses for Suicide Death.

|  |  | **Overall** | | | |  | | |
| --- | --- | --- | --- | --- | --- | --- | --- | --- |
|  | **Binary** | | |  |  | |  | |
|  | **n** | | **RR [95% CI]** |  |  | |  |  |
| **Pooled Effects** |  | |  |  |  | |  |  |
| **Specific Intervention Type** |  | |  |  |  | |  |  |
| Medication Only | 4 | | - |  |  | |  |  |
| Psychotherapy and Medication Combined | 1 | | - |  |  | |  |  |
| CT/CBT | 1 | | - |  |  | |  |  |
| Mixed Psychotherapy Modalities | 0 | | - |  |  | |  |  |
| Psychoeducation | 0 | | - |  |  | |  |  |
| DBT | 0 | | - |  |  | |  |  |
| Parenting Skills Training | 0 | | - |  |  | |  |  |
|  |  | |  |  |  | |  |  |
| **Control Group Type** |  | |  |  |  | |  |  |
| No Treatment | 0 | | - |  |  | |  |  |
| Placebo | 3 | | - |  |  | |  |  |
| Active Treatment | 3 | | - |  |  | |  |  |
|  |  | |  |  |  | |  |  |
| **Medication Class** |  | |  |  |  | |  |  |
| Selective Serotonin Reuptake Inhibitor | 4 | | - |  |  | |  |  |
| Atypical Antipsychotic | 0 | | - |  |  | |  |  |
| Selective Norepinephrine Reuptake Inhibitor | 0 | | - |  |  | |  |  |
| Alpha-2 Adrenergic Agonist | 0 | | - |  |  | |  |  |
| Hypnotic | 0 | | - |  |  | |  |  |
| Atypical Antidepressant | 0 | | - |  |  | |  |  |
| Mood Stabilizer | 0 | | - |  |  | |  |  |
|  |  | |  |  |  | |  |  |
| **Sample Severity** |  | |  |  |  | |  |  |
| General | 0 | | - |  |  | |  |  |
| Clinical | 6 | | 0.77 [0.47, 1.26] |  |  | |  |  |
| SITB | 0 | | - |  |  | |  |  |
|  |  | |  |  |  | |  |  |
| **Age Group** |  | |  |  |  | |  |  |
| Children | 0 | | - |  |  | |  |  |
| Adolescents | 6 | | 0.77 [0.47, 1.26] |  |  | |  |  |
|  |  | |  |  |  | |  |  |
| **Intervention Target Type** |  | |  |  |  | |  |  |
| SITBs | 0 | | - |  |  | |  |  |
| Psychopathology | 6 | | 0.77 [0.47, 1.26] |  |  | |  |  |
| Other |  | |  |  |  | |  |  |
|  |  | |  |  |  | |  |  |
| **Treatment Components** |  | |  |  |  | |  |  |
| Individual Only | 4 | | - |  |  | |  |  |
| Family Only | 0 | | - |  |  | |  |  |
| Group Only | 0 | | - |  |  | |  |  |
| Individual and Family | 2 | | - |  |  | |  |  |
| Individual, Family, and Group | 0 | | - |  |  | |  |  |
| School-based | 0 | | - |  |  | |  |  |
| Individual Skills Training Provided | 2 | | - |  |  | |  |  |
| Designed or Adapted for Adolescents | 2 | | - |  |  | |  |  |
|  |  | |  |  |  | |  |  |
| **Study Quality** |  | |  |  |  | |  |  |
| Weak | 2 | | - |  |  | |  |  |
| Moderate | 4 | | - |  |  | |  |  |
| Strong | 0 | | - |  |  | |  |  |
|  |  | |  |  |  | |  |  |
| **Therapist Training and Adherence** |  | |  |  |  | |  |  |
| Therapist Adherence Check | 5 | | 0.73 [0.45, 1.21] |  |  | |  |  |
| Therapist Pre-Treatment Training | 5 | | 0.73 [0.45, 1.21] |  |  | |  |  |

*Note.* Estimates were not reported for analyses involving fewer than five effect sizes to improve the reliability and accuracy of estimates. n = number of effect sizes, RR = weighted mean risk ratio, 95% CI = 95% confidence interval. Dashes indicate unavailable information. Bold indicates an effect estimate which is significantly different from pooled effects (i.e., nonoverlapping confidence intervals).

Table S4. Moderator analyses for NSSI.

|  |  | **Overall** | | | | | |
| --- | --- | --- | --- | --- | --- | --- | --- |
|  | **Binary** | | |  |  | **Continuous** | |
|  | **n** | | **RR [95% CI]** |  |  | **n** | ***g* [95% CI]** |
| **Pooled Effects** |  | |  |  |  |  |  |
| **Specific Intervention Type** |  | |  |  |  |  |  |
| Medication Only | 21 | | 1.11 [0.73, 1.68] |  |  | 0 | - |
| Psychotherapy and Medication Combined | 2 | | - |  |  | 0 | - |
| CT/CBT | 2 | | - |  |  | 1 | - |
| Mixed Psychotherapy Modalities | 0 | | - |  |  | 0 | - |
| Psychoeducation | 0 | | - |  |  | 0 | - |
| DBT | 1 | | - |  |  | 0 | - |
| Parenting Skills Training | 0 | | - |  |  | 0 | - |
|  |  | |  |  |  |  |  |
| **Control Group Type** |  | |  |  |  |  |  |
| No Treatment | 0 | | - |  |  | 0 | - |
| Placebo | 13 | | 1.07 [0.61, 1.85] |  |  | 0 | - |
| Active Treatment | 15 | | 1.23 [0.88, 1.72] |  |  | 1 | - |
|  |  | |  |  |  |  |  |
| **Medication Class** |  | |  |  |  |  |  |
| Selective Serotonin Reuptake Inhibitor | 4 | | - |  |  | 0 | - |
| Atypical Antipsychotic | 3 | | - |  |  | 0 | - |
| Selective Norepinephrine Reuptake Inhibitor | 11 | | 1.37 [0.83, 2.27] |  |  | 0 | - |
| Alpha-2 Adrenergic Agonist | 2 | | - |  |  | 0 | - |
| Hypnotic | 0 | | - |  |  | 0 | - |
| Atypical Antidepressant | 0 | | - |  |  | 0 | - |
| Mood Stabilizer | 0 | | - |  |  | 0 | - |
|  |  | |  |  |  |  |  |
| **Sample Severity** |  | |  |  |  |  |  |
| General | 0 | | - |  |  | 0 | - |
| Clinical | 26 | | 1.23 [0.90, 1.68] |  |  | 0 | - |
| SITB | 2 | | - |  |  | 1 | - |
|  |  | |  |  |  |  |  |
| **Age Group** |  | |  |  |  |  |  |
| Children | 1 | | - |  |  | 0 | - |
| Adolescents | 26 | | 1.13 [0.84, 1.51] |  |  | 1 | - |
|  |  | |  |  |  |  |  |
| **Intervention Target Type** |  | |  |  |  |  |  |
| SITBs | 8 | | 0.97 [0.62, 1.52] |  |  | 1 | - |
| Psychopathology | 20 | | 1.36 [0.94, 1.96] |  |  | 0 | - |
| Other | 0 | | - |  |  | 0 | - |
|  |  | |  |  |  |  |  |
| **Treatment Components** |  | |  |  |  |  |  |
| Individual Only | 24 | | 1.13 [0.82, 1.57] |  |  | 1 | - |
| Family Only | 0 | | - |  |  | 0 | - |
| Group Only | 0 | | - |  |  | 0 | - |
| Individual and Family | 4 | | - |  |  | 0 | - |
| Individual, Family, and Group | 0 | | - |  |  | 0 | - |
| School-based | 0 | | - |  |  | 0 | - |
| Individual Skills Training Provided | 6 | | 1.33 [0.83, 2.13] |  |  | 1 | - |
| Designed or Adapted for Adolescents | 6 | | 1.25 [0.80, 1.96] |  |  | 1 | - |
|  |  | |  |  |  |  |  |
| **Study Quality** |  | |  |  |  |  |  |
| Weak | 14 | | 1.23 [0.84, 1.80] |  |  | 1 | - |
| Moderate | 13 | | 1.13 [0.73, 1.74] |  |  | 0 | - |
| Strong | 1 | | - |  |  | 0 | - |
|  |  | |  |  |  |  |  |
| **Therapist Training and Adherence** |  | |  |  |  |  |  |
| Therapist Adherence Check | 6 | | 1.26 [0.85, 1.88] |  |  | 0 | - |
| Therapist Pre-Treatment Training | 5 | | 1.31 [0.88, 1.96] |  |  | 0 | - |

*Note.* Estimates were not reported for analyses involving fewer than five effect sizes to improve the reliability and accuracy of estimates. n = number of effect sizes, RR = weighted mean risk ratio, 95% CI = 95% confidence interval. Dashes indicate unavailable information. Bold indicates an effect estimate which is significantly different from pooled effects (i.e., nonoverlapping confidence intervals).

Table S5. Moderator analyses for Self-Harm.

|  |  | **Overall** | | | | | |
| --- | --- | --- | --- | --- | --- | --- | --- |
|  | **Binary** | | |  |  | **Continuous** | |
|  | **n** | | **RR [95% CI]** |  |  | **n** | ***g* [95% CI]** |
| **Pooled Effects** |  | |  |  |  |  |  |
| **Specific Intervention Type** |  | |  |  |  |  |  |
| Medication Only | 18 | | 1.24 [0.69, 2.23] |  |  | 0 | - |
| Psychotherapy and Medication Combined | 3 | | - |  |  | 0 | - |
| CT/CBT | 1 | | - |  |  | 0 | - |
| Mixed Psychotherapy Modalities | 4 | | - |  |  | 2 | - |
| Psychoeducation | 0 | | - |  |  | 0 | - |
| DBT | 1 | | - |  |  | 1 | - |
| Parenting Skills Training | 0 | | - |  |  | 0 | - |
|  |  | |  |  |  |  |  |
| **Control Group Type** |  | |  |  |  |  |  |
| No Treatment | 0 | | - |  |  | 2 | - |
| Placebo | 15 | | 1.09 [0.54, 2.20] |  |  | 0 | - |
| Active Treatment | 15 | | 0.98 [0.79, 1.22] |  |  | 5 | -0.01 [-0.24, 0.23] |
|  |  | |  |  |  |  |  |
| **Medication Class** |  | |  |  |  |  |  |
| Selective Serotonin Reuptake Inhibitor | 5 | | 1.78 [0.79, 3.99] |  |  | 0 | - |
| Atypical Antipsychotic | 7 | | 1.03 [0.34, 3.12] |  |  | 0 | - |
| Selective Norepinephrine Reuptake Inhibitor | 1 | | - |  |  | 0 | - |
| Alpha-2 Adrenergic Agonist | 0 | | - |  |  | 0 | - |
| Hypnotic | 0 | | - |  |  | 0 | - |
| Atypical Antidepressant | 3 | | - |  |  | 0 | - |
| Mood Stabilizer | 1 | | - |  |  | 0 | - |
|  |  | |  |  |  |  |  |
| **Sample Severity** |  | |  |  |  |  |  |
| General | 1 | | - |  |  | 0 | - |
| Clinical | 22 | | 1.15 [0.81, 1.65] |  |  | 3 | - |
| SITB | 7 | | 0.91 [0.71, 1.18] |  |  | 4 | - |
|  |  | |  |  |  |  |  |
| **Age Group** |  | |  |  |  |  |  |
| Children | 1 | | - |  |  | 0 | - |
| Adolescents | 29 | | 0.99 [0.81, 1.22] |  |  | 7 | 0.12 [-0.07, 0.32] |
|  |  | |  |  |  |  |  |
| **Intervention Target Type** |  | |  |  |  |  |  |
| SITBs | 6 | | 0.96 [0.70, 1.29] |  |  | 6 | 0.12 [-0.10, 0.34] |
| Psychopathology | 23 | | 0.88 [0.72, 1.07] |  |  | 1 | - |
| Other | 1 | | - |  |  | 0 | - |
|  |  | |  |  |  |  |  |
| **Treatment Components** |  | |  |  |  |  |  |
| Individual Only | 20 | | 0.99 [0.68, 1.43] |  |  | 0 | - |
| Family Only | 0 | | - |  |  | 0 | - |
| Group Only | 4 | | - |  |  | 3 | - |
| Individual and Family | 5 | | 0.77 [0.57, 1.05] |  |  | 0 | - |
| Individual, Family, and Group | 1 | | - |  |  | 4 | - |
| School-based | 0 | | - |  |  | 0 | - |
| Individual Skills Training Provided | 9 | | 1.07 [0.83, 1.38] |  |  | 7 | 0.12 [-0.07, 0.32] |
| Designed or Adapted for Adolescents | 11 | | 0.97 [0.75, 1.24] |  |  | 7 | 0.12 [-0.07, 0.32] |
|  |  | |  |  |  |  |  |
| **Study Quality** |  | |  |  |  |  |  |
| Weak | 7 | | **0.78 [0.63, 0.98]** |  |  | 2 | - |
| Moderate | 21 | | 1.08 [0.83, 1.39] |  |  | 2 | - |
| Strong |  | |  |  |  |  |  |
|  |  | |  |  |  |  |  |
| **Therapist Training and Adherence** |  | |  |  |  |  |  |
| Therapist Adherence Check | 11 | | 0.96 [0.77, 1.20] |  |  | 7 | 0.12 [-0.07, 0.32] |
| Therapist Pre-Treatment Training | 11 | | 0.96 [0.77, 1.20] |  |  | 7 | 0.12 [-0.07, 0.32] |

*Note.* Estimates were not reported for analyses involving fewer than five effect sizes to improve the reliability and accuracy of estimates. n = number of effect sizes, RR = weighted mean risk ratio, 95% CI = 95% confidence interval. Dashes indicate unavailable information. Bold indicates an effect estimate which is significantly different from pooled effects (i.e., nonoverlapping confidence intervals).

Table S6. Moderator analyses for Hospitalization.

|  |  | **Overall** | | | | | |
| --- | --- | --- | --- | --- | --- | --- | --- |
|  | **Binary** | | |  |  | **Continuous** | |
|  | **n** | | **RR [95% CI]** |  |  | **n** | ***g* [95% CI]** |
| **Pooled Effects** |  | |  |  |  |  |  |
| **Specific Intervention Type** |  | |  |  |  |  |  |
| Medication Only | 1 | | - |  |  | 0 | - |
| Psychotherapy and Medication Combined | 0 | | - |  |  | 0 | - |
| CT/CBT | 2 | | - |  |  | 0 | - |
| Mixed Psychotherapy Modalities | 0 | | - |  |  | 0 | - |
| Psychoeducation | 0 | | - |  |  | 0 | - |
| DBT | 2 | | - |  |  | 0 | - |
| Parenting Skills Training | 0 | | - |  |  | 0 | - |
|  |  | |  |  |  |  |  |
| **Control Group Type** |  | |  |  |  |  |  |
| No Treatment | 0 | | - |  |  | 0 | - |
| Placebo | 1 | | - |  |  | 0 | - |
| Active Treatment | 7 | | 1.12 [0.90, 1.40] |  |  | 0 | - |
|  |  | |  |  |  |  |  |
| **Medication Class** |  | |  |  |  |  |  |
| Selective Serotonin Reuptake Inhibitor | 1 | | - |  |  | 0 | - |
| Atypical Antipsychotic | 0 | | - |  |  | 0 | - |
| Selective Norepinephrine Reuptake Inhibitor | 0 | | - |  |  | 0 | - |
| Alpha-2 Adrenergic Agonist | 0 | | - |  |  | 0 | - |
| Hypnotic | 0 | | - |  |  | 0 | - |
| Atypical Antidepressant | 0 | | - |  |  | 0 | - |
| Mood Stabilizer | 0 | | - |  |  | 0 | - |
|  |  | |  |  |  |  |  |
| **Sample Severity** |  | |  |  |  |  |  |
| General | 0 | | - |  |  | 0 | - |
| Clinical | 4 | | - |  |  | 1 | - |
| SITB | 4 | | - |  |  | 0 | - |
|  |  | |  |  |  |  |  |
| **Age Group** |  | |  |  |  |  |  |
| Children | 0 | | - |  |  | 0 | - |
| Adolescents | 8 | | 1.11 [0.89, 1.39] |  |  | 1 | - |
|  |  | |  |  |  |  |  |
| **Intervention Target Type** |  | |  |  |  |  |  |
| SITBs | 4 | | - |  |  | 0 | - |
| Psychopathology | 4 | | - |  |  | 1 | - |
| Other | 0 | | - |  |  | 0 | - |
|  |  | |  |  |  |  |  |
| **Treatment Components** |  | |  |  |  |  |  |
| Individual Only | 1 | | - |  |  | 0 | - |
| Family Only | 2 | | - |  |  | 0 | - |
| Group Only | 0 | | - |  |  | 0 | - |
| Individual and Family | 3 | | - |  |  | 0 | - |
| Individual, Family, and Group | 2 | | - |  |  | 1 | - |
| School-based | 0 | | - |  |  | 0 | - |
| Individual Skills Training Provided | 5 | | 0.65 [0.24, 1.72] |  |  | 1 | - |
| Designed or Adapted for Adolescents | 7 | | 1.12 [0.90, 1.40] |  |  | 1 | - |
|  |  | |  |  |  |  |  |
| **Study Quality** |  | |  |  |  |  |  |
| Weak | 6 | | 0.74 [0.29, 1.87] |  |  | 1 | - |
| Moderate | 2 | | - |  |  | 0 | - |
| Strong | 0 | | - |  |  | 0 | - |
|  |  | |  |  |  |  |  |
| **Therapist Training and Adherence** |  | |  |  |  |  |  |
| Therapist Adherence Check | 7 | | 1.12 [0.90, 1.40] |  |  | 1 | - |
| Therapist Pre-Treatment Training | 7 | | 1.12 [0.90, 1.40] |  |  | 1 | - |

*Note.* Estimates were not reported for analyses involving fewer than five effect sizes to improve the reliability and accuracy of estimates. n = number of effect sizes, RR = weighted mean risk ratio, 95% CI = 95% confidence interval. Dashes indicate unavailable information. Bold indicates an effect estimate which is significantly different from pooled effects (i.e., nonoverlapping confidence intervals).

Table S7. Moderator analyses for Other/Combined SITBs.

|  |  | **Overall** | | | | | |
| --- | --- | --- | --- | --- | --- | --- | --- |
|  | **Binary** | | |  |  | **Continuous** | |
|  | **n** | | **RR [95% CI]** |  |  | **n** | ***g* [95% CI]** |
| **Pooled Effects** |  | |  |  |  |  |  |
| **Specific Intervention Type** |  | |  |  |  |  |  |
| Medication Only | 82 | | **1.40 [1.15, 1.70]** |  |  | 0 | - |
| Psychotherapy and Medication Combined | 16 | | 0.94 [0.73, 1.22] |  |  | 0 | - |
| CT/CBT | 7 | | 0.99 [0.42, 2.32] |  |  | 2 | - |
| Mixed Psychotherapy Modalities | 0 | | - |  |  | 1 | - |
| Psychoeducation | 0 | | - |  |  | 0 | - |
| DBT | 0 | | - |  |  | 0 | - |
| Parenting Skills Training | 0 | | - |  |  | 1 | - |
|  |  | |  |  |  |  |  |
| **Control Group Type** |  | |  |  |  |  |  |
| No Treatment | 2 | | - |  |  | 1 | - |
| Placebo | 76 | | **1.59 [1.30, 1.94]** |  |  | 0 | - |
| Active Treatment | 33 | | 0.76 [0.58, 1.00] |  |  | 3 | - |
|  |  | |  |  |  |  |  |
| **Medication Class** |  | |  |  |  |  |  |
| Selective Serotonin Reuptake Inhibitor | 49 | | 1.41 [1.12, 1.78] |  |  | 0 | - |
| Atypical Antipsychotic | 11 | | 0.67 [0.31, 1.45] |  |  | 0 | - |
| Selective Norepinephrine Reuptake Inhibitor | 17 | | 1.27 [0.83, 1.92] |  |  | 0 | - |
| Alpha-2 Adrenergic Agonist | 0 | | - |  |  | 0 | - |
| Hypnotic | 0 | | - |  |  | 0 | - |
| Atypical Antidepressant | 2 | | - |  |  | 0 | - |
| Mood Stabilizer | 1 | | - |  |  | 0 | - |
|  |  | |  |  |  |  |  |
| **Sample Severity** |  | |  |  |  |  |  |
| General | 3 | | **-** |  |  | 0 | - |
| Clinical | 106 | | 1.20 [1.01, 1.43] |  |  | 1 | - |
| SITB | 2 | | - |  |  | 3 | - |
|  |  | |  |  |  |  |  |
| **Age Group** |  | |  |  |  |  |  |
| Children | 4 | | - |  |  | 0 | - |
| Adolescents | 107 | | 1.19 [1.00, 1.41] |  |  | 4 | - |
|  |  | |  |  |  |  |  |
| **Intervention Target Type** |  | |  |  |  |  |  |
| SITBs | 9 | | 0.92 [0.74, 1.13] |  |  | 3 | - |
| Psychopathology | 101 | | 1.22 [1.03, 1.46] |  |  | 1 | - |
| Other | 1 | | - |  |  | 0 | - |
|  |  | |  |  |  |  |  |
| **Treatment Components** |  | |  |  |  |  |  |
| Individual Only | 88 | | 1.30 [1.08, 1.58] |  |  | 1 | - |
| Family Only | 1 | | - |  |  | 2 | - |
| Group Only | 0 | | - |  |  | 0 | - |
| Individual and Family | 19 | | 1.14 [0.77, 1.68] |  |  | 1 | - |
| Individual, Family, and Group | 0 | | - |  |  | 0 | - |
| School-based | 3 | | - |  |  | 0 | - |
| Individual Skills Training Provided | 21 | | 1.06 [0.72, 1.57] |  |  | 2 | - |
| Designed or Adapted for Adolescents | 22 | | 1.04 [0.67, 1.61] |  |  | 4 | - |
|  |  | |  |  |  |  |  |
| **Study Quality** |  | |  |  |  |  |  |
| Weak | 74 | | 1.26 [1.02, 1.55] |  |  | 2 | - |
| Moderate | 35 | | 1.05 [0.80, 1.37] |  |  | 2 | - |
| Strong | 2 | | - |  |  | 0 | - |
|  |  | |  |  |  |  |  |
| **Therapist Training and Adherence** |  | |  |  |  |  |  |
| Therapist Adherence Check | 22 | | 0.78 [0.55, 1.11] |  |  | 3 | - |
| Therapist Pre-Treatment Training | 18 | | 0.75 [0.52, 1.08] |  |  | 2 | - |

*Note.* Estimates were not reported for analyses involving fewer than five effect sizes to improve the reliability and accuracy of estimates. n = number of effect sizes, RR = weighted mean risk ratio, 95% CI = 95% confidence interval. Dashes indicate unavailable information. Bold indicates an effect estimate which is significantly different from pooled effects (i.e., nonoverlapping confidence intervals).
