# Supplementary material for: Efficacy of interventions for suicide and self-injury in children and adolescents: a meta-analysis
Source: Sci Rep. 2022 Jul 19;12:12313. doi: 10.1038/s41598-022-16567-8 (PMC9296501; doi:10.1038/s41598-022-16567-8)
Supplement: Supplementary file 4 — Supplementary Information 2. [file 41598_2022_16567_MOESM4_ESM.docx]

**Supplement 4**

**Post-Hoc Power Analysis**

To assess the possibility that low statistical power may have influenced our results, we conducted a post-hoc power analysis. To avoid common pitfalls of retrospective power analyses (Hoenig & Heisey, 2001), we adhered to guidelines which recommend basing retrospective power computations on the smallest *meaningful* effect size (Valentine et al., 2010). This method takes into account the observed number of studies, within-study sample sizes, and between-study heterogeneity, but does not assume that population parameters are equivalent to the observed sample statistics. Given the weak effects detected in this study’s parent meta-analysis (Fox et al., 2020), we reasoned that a risk ratio of 0.95 (representing a 5% reduction in binary outcomes) and a standardized mean difference of 0.10 (representing a “very small” treatment effect for continuous outcomes) were appropriately conservative estimates for a meaningful effect of treatment. Our results revealed that we were powered at >99.99% to detect a 5% reduction in binary outcomes. We were powered at 86.12% to detect a “very small” treatment effect of 0.10 for continuous outcomes and at 99.99% to detect a "small" treatment effect of 0.20.
