# Supplementary material for: Efficacy of interventions for suicide and self-injury in children and adolescents: a meta-analysis
Source: Sci Rep. 2022 Jul 19;12:12313. doi: 10.1038/s41598-022-16567-8 (PMC9296501; doi:10.1038/s41598-022-16567-8)
Supplement: Supplementary file 5 — Supplementary Information 3. [file 41598_2022_16567_MOESM5_ESM.docx]

**Supplement 5**

Because meta-analytic findings are prone to both Type I and Type II error [1], we conducted Trial Sequential Analysis (TSA) to reduce the likelihood of spurious conclusions. Findings from TSA of pooled binary SITB outcomes are displayed in Figure S1, and findings for pooled continuous SITB outcomes are displayed in Figure S2. TSA was performed using Trial Sequential analysis software [2], and our interpretations were informed by guidelines delineated by Wetterslev and colleagues [1, 3] and descriptions provided by Odor and colleagues [4].


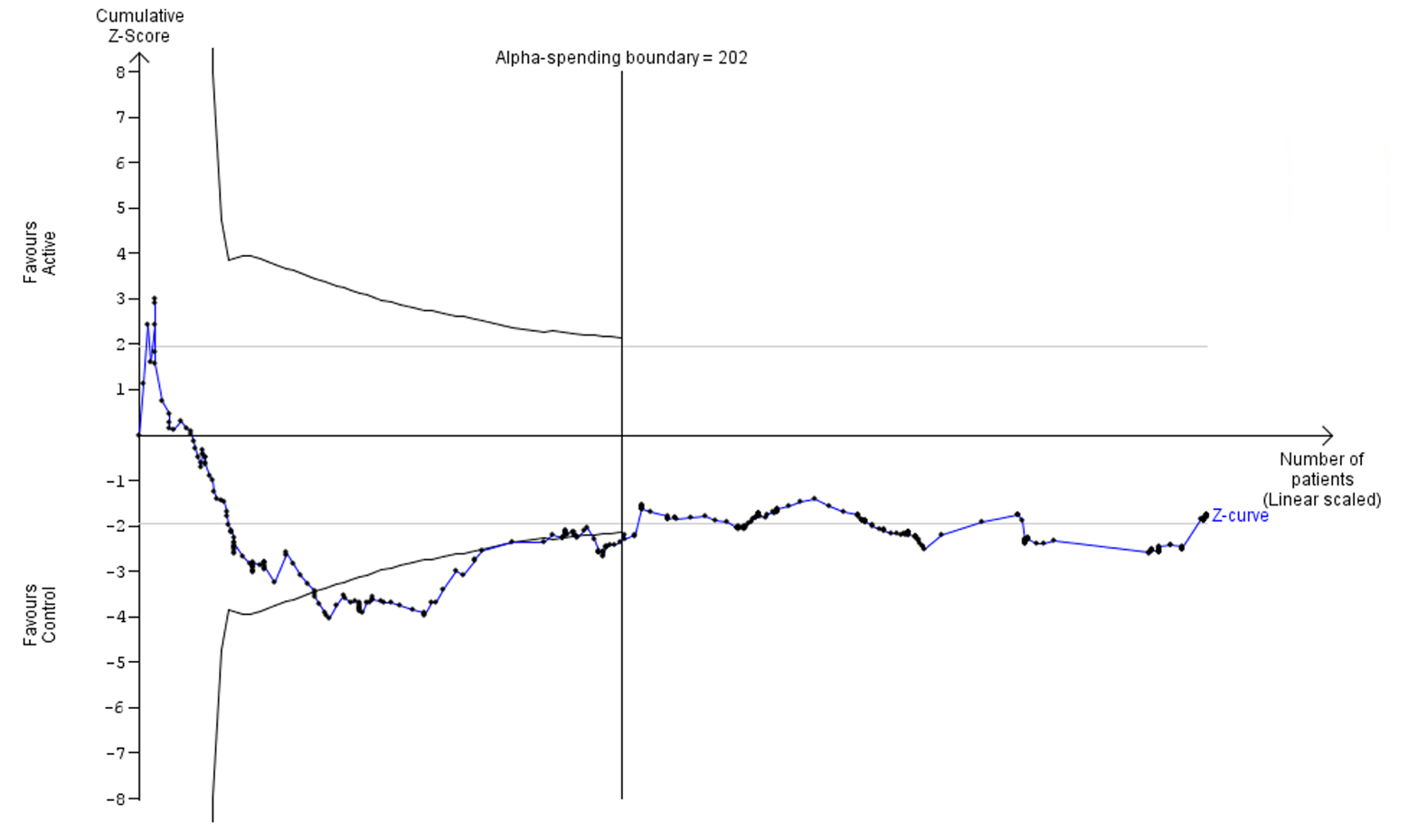


**Figure S1**. Trial Sequential Analysis of Pooled Binary SITB Outcomes. The horizontal lines at +1.96 and −1.96 represent conventional confidence intervals (i.e., α = 5%). In TSA analysis, “firm evidence” has been reached when the cumulative z-curve (the blue line, with each consecutive RCT represented by a black circle) crosses the calculated trial sequential monitoring boundaries (TSMBs; the curved symmetrical lines above and below the y-axis) before the calculated information size (IS; the vertical line) is reached. Spurious significant differences between treatments arise when the cumulative z-curve crosses the conventional confidence intervals without crossing the calculated TSMBs. Our results demonstrate that the minimum required IS has been reached. The cumulative z-score curve crossed the calculated TSMBs before the IS was reached, indicating superiority of the control arm over the active arm of evaluated interventions. In other words, results from TSA of pooled binary SITB outcomes yielded evidence suggesting statistically significant, but potentially iatrogenic, effects of evaluated interventions.


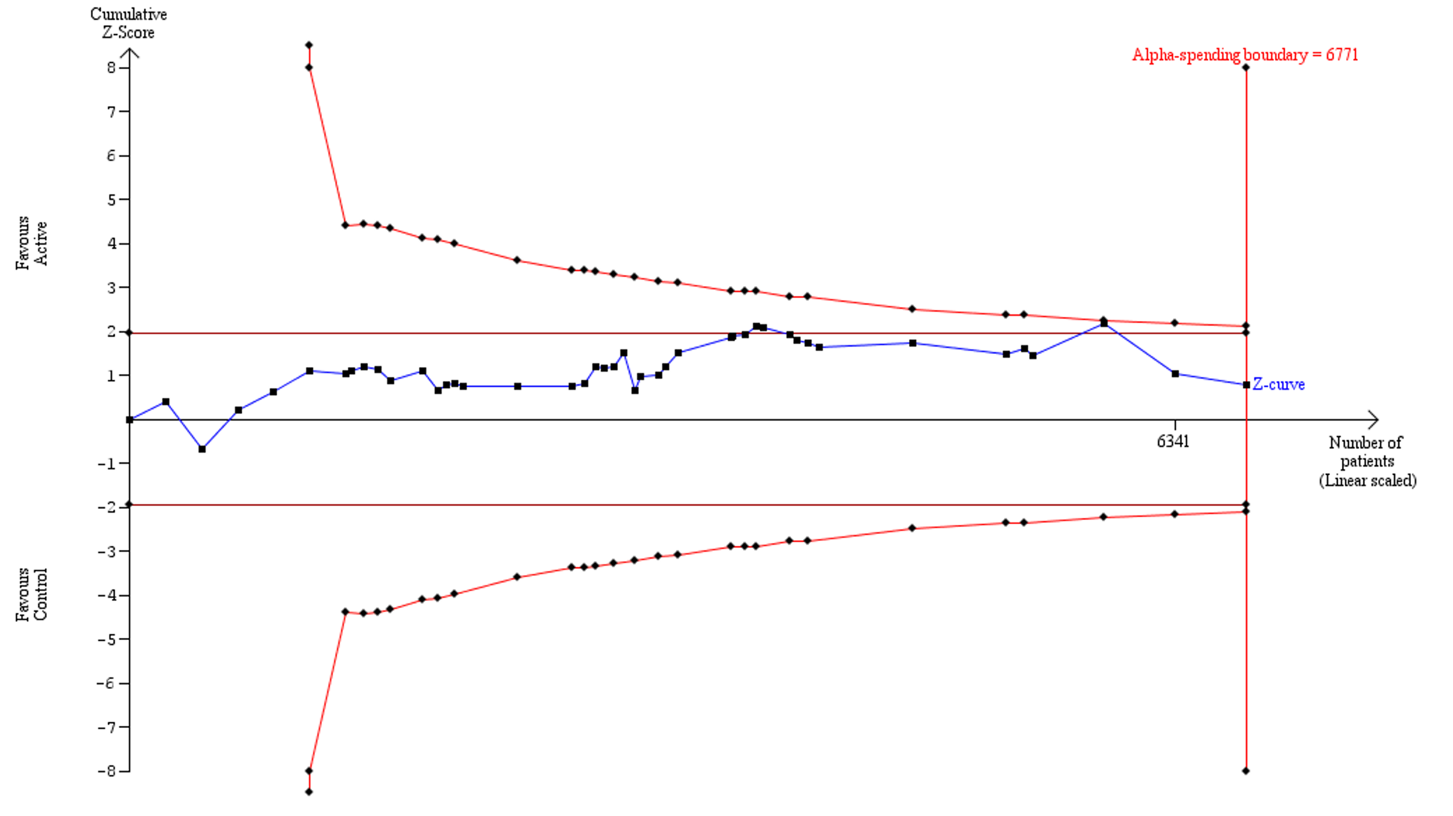


**Figure S2**. Trial Sequential Analysis of Pooled Continuous SITB Outcomes. Results from pooled analyses of continuous outcomes demonstrate that the minimum required IS has been reached. The cumulative z-score curve does not cross the calculated TSMBs before the IS was reached, indicating that our conventional random-effects meta-analysis could have produced a potentially spurious result. However, because we did not initially detect statistically significant treatment effects, these results are aligned with our findings that there were no statistically significant differences between treatment conditions and control conditions on pooled continuous SITB outcomes.

**References**

[1] Wetterslev J, Jakobsen JC, Gluud C. Trial Sequential Analysis in systematic reviews with meta-analysis. *BMC Med Res Methodol* 2017; 17: 39.

[2] Thorlund K, Engstrom J, Wetterslev J, et al. *Trial Sequential Analysis*. Copenhagen Trial Unit, 2017.

[3] Wetterslev J, Thorlund K, Brok J, et al. Trial sequential analysis may establish when firm evidence is reached in cumulative meta-analysis. *J Clin Epidemiol* 2008; 61: 64–75.

[4] Odor PM, Bampoe S, Gilhooly D, et al. Perioperative interventions for prevention of postoperative pulmonary complications: systematic review and meta-analysis. *BMJ* 2020; m540.
